# Pain mechanisms in the transgender individual: a review

**DOI:** 10.3389/fpain.2024.1241015

**Published:** 2024-03-27

**Authors:** Jennifer T. Anger, Laura K. Case, Andrew P. Baranowski, Ardin Berger, Rebecca M. Craft, Lyn Ann Damitz, Rodney Gabriel, Tracy Harrison, Kirsten Kaptein, Sanghee Lee, Anne Z. Murphy, Engy Said, Stacey Abigail Smith, David A. Thomas, Maria del C. Valdés Hernández, Victor Trasvina, Ursula Wesselmann, Tony L. Yaksh

**Affiliations:** ^1^ Department of Urology, University of California San Diego, San Diego, CA, United States; ^2^ Department of Anesthesiology, University of California San Diego, San Diego, CA, United States; ^3^ Pelvic Pain Medicine and Neuromodulation, University College Hospital Foundation Trust, University College London, London, United Kingdom; ^4^ Anesthesiology, Perioperative and Pain Medicine, Stanford University, Stanford, CA, United States; ^5^ Department of Psychology, Washington State University, Pullman, WA, United States; ^6^ Division of Plastic and Reconstructive Surgery, University of North Carolina, Chapel Hill, NC, United States; ^7^ Division of Regional Anesthesia, University of California San Diego, San Diego, CA, United States; ^8^ Department of OB/GYN & Reproductive Sciences, University of California San Diego, San Diego, CA, United States; ^9^ Division of Plastic Surgery, University of California San Diego, San Diego, CA, United States; ^10^ Neuroscience Institute, Georgia State University, Atlanta, GA, United States; ^11^ Division of Infection Disease, The Hope Clinic of Emory University, Atlanta, GA, United States; ^12^ Office of Research on Women's Health, National Institutes of Health, Bethesda, MD, United States; ^13^ Department of Neuroimaging Sciences, Center for Clinical Brain Sciences, University of Edinburgh, Edinburgh, United Kingdom; ^14^ Departments of Anesthesiology and Perioperative Medicine/Division of Pain Medicine, Neurology and Psychology, and Consortium for Neuroengineering and Brain-Computer Interfaces, The University of Alabama at Birmingham, Birmingham, AL, United States

**Keywords:** transgender (GLBT) issues, post operative pain, gender binary system, hormone replacement therapy (HRT), gender affirming care

## Abstract

**Specific Aim:**

Provide an overview of the literature addressing major areas pertinent to pain in transgender persons and to identify areas of primary relevance for future research.

**Methods:**

A team of scholars that have previously published on different areas of related research met periodically though zoom conferencing between April 2021 and February 2023 to discuss relevant literature with the goal of providing an overview on the incidence, phenotype, and mechanisms of pain in transgender patients. Review sections were written after gathering information from systematic literature searches of published or publicly available electronic literature to be compiled for publication as part of a topical series on gender and pain in the Frontiers in Pain Research.

**Results:**

While transgender individuals represent a significant and increasingly visible component of the population, many researchers and clinicians are not well informed about the diversity in gender identity, physiology, hormonal status, and gender-affirming medical procedures utilized by transgender and other gender diverse patients. Transgender and cisgender people present with many of the same medical concerns, but research and treatment of these medical needs must reflect an appreciation of how differences in sex, gender, gender-affirming medical procedures, and minoritized status impact pain.

**Conclusions:**

While significant advances have occurred in our appreciation of pain, the review indicates the need to support more targeted research on treatment and prevention of pain in transgender individuals. This is particularly relevant both for gender-affirming medical interventions and related medical care. Of particular importance is the need for large long-term follow-up studies to ascertain best practices for such procedures. A multi-disciplinary approach with personalized interventions is of particular importance to move forward.

## Background

The intersection of sex and pain has become a focus of extensive research at the clinical and preclinical level. Numerous sex differences exist in pain and its processing in humans (1) However, pain research frequently conflates sex and gender. Sex refers to biological aspects of maleness or femaleness. It is a complex label used to describe the totality of distinguishable characteristics of an organism, including its reproductive structure, functions, phenotype, and genotype (2, 3). On the other hand, gender refers to the psychological, behavioral, social, and cultural aspects of being male, female, or another gender identity. Expectations of gender differ by culture. However, gender is frequently assumed on the basis of sex assigned at birth; a difference in gender identity from that assumed/assigned at birth is broadly referred to as being transgender (1). The pain experience of transgender individuals has been poorly studied.

There are several reasons why pain experience may differ in individuals who are transgender (trans). One reason is the stress that many trans individuals experience due to a frequently hostile social and political environment (4, 5). Many trans individuals also experience gender dysphoria, defined as the distress and emotional discomfort from their gender incongruence, which is created and/or exacerbated by a societal environment that does not consistently accept gender diversity (6, 7). Social marginalization is a frequent risk factor for suicidality (4). Indeed, a large study of transgender adults in the United States, 40% endorsed a lifetime suicide attempt (8). The compounding effects of distress generated by gender dysphoria is also reflected in the high incidence of suicide and disabling behavioral syndromes in transgender individuals in whom the dysphoria is not addressed (4, 9, 10). As will be reviewed below, it is widely appreciated in many human conditions that such unmitigated stressors can lead to profound exacerbations of pain phenotypes (11, 12) and this may have a strong sex/gender component (13, 14).

Other differences in biology relevant to pain perception may arise from gender-affirming clinical interventions. These interventions may involve hormonal or surgical therapies to assist in modifying a person's primary and secondary sexual characteristics. Such interventions can significantly impact the psychological and physiological components of the pain experience (15, 16). These variables suggest the complexity of pain expression in the increasingly visible and diverse population of transgender adults and youth, along with the appreciation that transgender patients represent a medically underserved, stigmatized population (6). This has led the Institute of Medicine to identify transgender health as a research priority (17). However, the landmark report of the Institute of Medicine on pain published in the same year, did not address pain in transgender patients (18), and there is an urgent medical need to consider this important and understudied topic.

### Overarching aims of the review

The aim of this review is to summarize key literature, addressing variables related to pain in transgender individuals. We review the background and note issues related to categorization of gender identity. Specifically, we: (i) highlight the impact of discrimination, life experiences, and hormonal and surgical treatments on pain perception; (ii) consider aspects of the biology of neural processing of pain and potential differences in cis and trans individuals; (iii) consider the contribution of preclinical studies to understanding of the nexus of sex, gender, and biology. We also address the role of the NIH in supporting issues pertinent to pain in transgender individuals, and directions for future research. The breadth of the topic precluded an in-depth review of each of the topical areas, but it is our aim to address pivotal issues for which we feel information or answers are not currently available and as such, point to future directions of research. As a prelude to this discussion, it is important to appreciate the complex interplay between medical issues and associated pain issues that pertain to transgender patients. A comprehensive listing of relevant concepts is presented in the Appendix (Supplementary Table S1) of this paper.

### Search strategies

A group of twenty-one participants formed the core of the *ad hoc* study group (*Pain in Transgender Patients*). This group met periodically though zoom conferencing between August 2021 and February 2023. This group created a series of specific topics that they considered to reflect essential issues relevant to the question of pain in transgender patients. These topical areas were assigned to individuals who have expertise in the respective areas. Each created a series of topical paragraphs that drew from the published and publicly available literature. These paragraphs were compiled and editorially revised. Compiled drafts were redistributed to the core group for revisions and corrections. The final drafts were approved and submitted.

## Transgender patient populations

### Population estimates

As noted, gender-minoritized persons are an often marginalized populations in the health system (19). Current electronic health records in many health organizations are not positioned or equipped to store and share gender-related information appropriately (20). Further estimates of the size of the transgender population are complicated, as gender diverse individuals do not represent a homogeneous population. Broadly speaking, the World Professional Association for Transgender Health (WPATH) Standards of Care defines gender nonconformity or incongruence as “the extent to which a person's gender identity, role, or expression differs from the cultural norms prescribed for people of a particular sex” (21). Gender incongruence was previously coded in the International Statistical Classification of Diseases and Related Health Problems of the World Health Organization (WHO) as a ‘mental disorder’. In the new International Classification of Diseases and Related Health Problems (ICD-11), gender incongruence is coded under ’sexual health’ (22, 23). This replaces previous diagnostic categories such as “transsexualism” and “gender identity disorder of children” with “gender incongruence of adolescence and adulthood” and “gender incongruence of childhood”.

A global survey in 2021 noted that 2% of respondents from 27 countries identified as transgender, non-binary/non-conforming, or gender fluid (24). The *Behavioral Risk Factor Surveillance System* (BRFSS) telephone survey in the US estimates that adult transgender population range from 0.3% to 0.8% of the population across several states (25, 26), whereas a review of population-based surveys (2006–2016) found that 0.39% of respondents identified as transgender when this was an option (27). Transgender self-identification appears more common among younger age groups (28). This may relate to decreases on stigma and increased availability of services for trans individuals, as well as changes in categories of gender.

### Issues related to data collection in relation to gender identity

The numbers quoted above, often used to estimate population characteristics, have been challenged as underestimates for several reasons (29). First, transgender identity is not often ascertained in many federal census or national health surveys or is too narrowly defined. Lett and Everhart (30) have introduced the use of other sources to address transgender identity. These include the *Youth Risk Behavior Surveillance System* (31), the *National Crime Victimization Survey* (29), the *General Social Survey* (32), and the *National HIV Behavioral Surveillance System* (33). Second, determining gender identity through survey questions depends upon self-identification. Responders may not identify themselves as transgender on surveys, despite expressing a gender that is different than their gender assigned at birth (30). Third, a simple question and a one-step query methodology to determine transgender identity may misclassify transgender people (30).

The absence of inclusive concepts, terms, and codes for gender, sex, and sexual orientation has contributed to the inability to correctly identify sex and gender minorities in data sets and electronic health records. As a result, we can express only modest certainty about and the prevalence of transgender identity and more specifically about specific health needs and long-term health outcomes among transgender individuals (34). Practically, the prevalence of transgender identity has been defined by physical alterations as with medical or surgical transitions (35–37). However, as will be reviewed below, while useful and important measures, the complexity of gender identity, the effects of hormonal therapies and the impact of the societal milieu contributing to gender dysphoria, can complicate the linkage of these physical characteristics with gender assignment, and there is often little discussion considering general implementation of such systems alongside self-identification (38, 39).

An important element in advancing our understanding of the conditions and issues faced by transgender populations is the development of gender-appropriate data collection tools and healthcare records. It must be recognized that approaches employing International Classification of Diseases (ICD) codes or simple text linked to trans-related procedures prioritizes populations of individuals who exhibit higher utilization of gender-affirming therapies/surgeries, often with better insurance coverage and lower poverty rates (2). For these reasons, it has been argued that the gold standard for gender assignment in patients is self-identification (21).

## Pain in relation to gender identity

Differences in pain sensitivity/responsivity and incidence in cisgender (cis) men and women groups are well documented, wherein it is reported that women often display greater sensitivity than men (5, 40). Such differences are frequently noted in specific syndromes, such as migraine (41) musculoskeletal pain (41, 42), temporomandibular joint syndromes (43, 44), fibromyalgia (45, 46); rheumatoid, osteo- and psoriatic arthritis (47–49); and Sjögren's disease (50). Some of these pain syndromes, such as migraine headaches, are more frequent in cis women, and often overlap with other pain conditions (51). Other pain syndromes, such as cluster headache, as more frequent in cis men (52). Furthermore, several studies have been conducted to highlight how gender biases in pharmacological and non-pharmacological pain management differ in cismen and ciswomen. Although significant differences were found, the data often depended on both the treatment type and pain characteristics. However, the etiology of these differences is complex given differential treatment of men and women in pain (5).

While there are differences in pain between cis men and women, potential differences in trans individuals are much less well characterized, and systematic work is scant. Survey-based studies have suggested a higher prevalence of pain symptoms among transgender people compared to the general population (53), a difference which appears to be more pronounced for trans men as compared to cis men than between trans women and cis women (53–55). Aloisi and colleagues studied transgender women and men who had undergone hormonal treatment for at least one year, and retrospectively report whether pain had changed from before to after treatment. Approximately 30% of transgender women reported painful conditions, and 80% of those stated that these painful conditions started after the onset of hormone treatment (55). In contrast, approximately 60% of transgender men reported pain, but nearly half of the participants reported that pain lessened after the onset of hormone treatment (55). However, these studies included very small sample sizes, and further study with much larger samples is needed to confirm these potential differences

In a study involving 51 African-American cis and transgender individuals living with HIV and chronic pain, Strath and colleagues (56) reported that trans women and cis women demonstrated a greater temporal summation for heat pain stimuli or stimulation compared to cisgender men. Trans women showed greater mechanical summation of the sensation produced by repeated mechanical nociceptive stimulation than either cis women or men. Similarly, trans and cis women reported greater chronic pain severity as compared to cis men. Migraine prevalence in trans women taking gender affirming hormone therapy was similar to that of cis women in population estimates (57).

Many trans individuals undergo painful clinical interventions in gender affirmation protocols, including hormone replacement and surgeries (58–60). However, the impact of these interventions on pain may differ by gender. A recent systematic review examining the literature on gender minority health provides little information specifically related to pain (61). Pain has been indicated as one of the monitoring parameters for feminizing hormone therapy (62, 63). A study of 47 transgender women and 26 transgender men reported that up to 25% of trans women developed chronic pain with estrogen and/or anti-androgen therapy whereas 60% of trans men experienced significant improvement in chronic headache along with testosterone treatment (55). Trans women treated with estrogen display a prevalence of migraine similar to that of cis women, including aura (57, 64, 65). In contrast, a study that recruited 115 transgender individuals from a specialized clinic in Israel reported a high prevalence of fibromyalgia symptoms among transgender individuals in general and in transgender men in particular as compared to transgender women (53). A variety of rheumatic pathologies are known to have a strong immune mediated component, and sexual dimorphism occurs in innate and adaptive immunity and contributes to differences in the prevalence of rheumatic diseases (66). A recent review commented on the limited literature on arthritis among trans individuals but noted that, though the number of cases was only 14, eleven cases of such immune mediated disorders occur in trans women (67). In parallel with the functional significance of immune cells on pain mechanisms, there is a greater predisposition to chronic pain among individuals lacking a Y chromosome (68, 69). However, gender identity in transgender patients may play as important a role in the pain experience as the genetics associated with sex (56).

## Medical care for transgender pain patients

A prospective survey found that 90% of pediatric anesthesiologists (*n* = 374) were comfortable interacting with transgender patients but only 50% of the respondents felt equipped with the knowledge to manage the surgical needs of transgender patients (70). This and other studies (70–72) demonstrate a fundamental need to understand pain mechanisms in the pediatric transgender population and to provide specific training for clinical care teams including anesthesiologists. In this regard, Standards of Care are issued and updated by WPATH (World Professional Association for Transgender Health), providing health care guidelines for clinical outcomes in target populations including transgender individuals (73). Implementation of questions about pain perception and its burden in transgender populations would provide detailed information that includes, but is not limited to, age, medical conditions, hormonal or surgical status, all of which may enhance our understanding of pain in relation to gender. Furthermore, the shifting paradigm in transgender and gender-diverse populations needs to be embedded in health care (2). Approaches to pain management should consider the significant pharmacological and surgical interventions that many transgender individuals receive. In the following section we provide an overview of these interventions and their implications.

### Gender affirming surgical interventions

Transgender patients may undergo a range of gender-affirming surgical interventions (74). Procedures often thought of as feminizing may include breast augmentation, facial feminization (75–78), and gender-affirming pelvic surgery (GAPS), or “bottom” surgery, which usually includes a penectomy (removing the penis), bilateral orchiectomy (removing the testicles), vaginoplasty (creating a vagina) (49, 79), clitoroplasty (creating a clitoris from the penile glans), and labiaplasty (creating labia minora and majora). Shallow depth vaginoplasty, or vulvoplasty, is an alternative to full-depth vaginoplasty for women who do not wish to engage in penetrating vaginal intercourse (80).

Procedures often thought of as masculinizing may include mastectomy (chest or “top” surgery), masculinizing facial surgery, and masculinizing “bottom” surgery which may involve a hysterectomy with or without salpingo-oophorectomy (removal of fallopian tubes and ovaries), vaginectomy (removing the vaginal canal), phalloplasty (creation of a phallus using a pedicle-based flap), metoidioplasty (creating a smaller penis from the clitoris after it is elongated with testosterone therapy), and/or scrotoplasty (creation of a scrotum from labia majora).

Many trans individuals also undergo vocal cord surgeries including thyroplasty for transgender men, or glottoplasty for transgender women (78, 81–83). These interventions are rarely performed for transgender people under the age of 18 (84, 85) and are never performed under the age of 16, despite prevalent misconception.

Gender-affirming medical and surgical treatments can increase positive body image in trans individuals by aligning bodily appearance with gender and desired sex, as a study on transgender individuals in Zurich found (86). Gender affirming hormone therapies have also led to an increase in body image and self-esteem (87). Similarly, hair removal procedures (desired by nearly 90% of transgender people) were associated with significant mental health benefits (88, 89).

In a comprehensive literature review, several insights were provided regarding gender affirming surgery (GAS) and trends in its use: (i) GAS is more common in transgender men than in transgender women, and least common in gender non-binary or nonconforming populations; (ii) Genital procedures are less common than chest surgery, with prevalence rates for genital procedures being about 25%–50% for transgender men and 5%–10% for transgender women (28, 90, 91). Gender-affirming mastectomy, or “top” surgery, is a common procedure for trans men. The demand for top surgery by transmen has increased 15% since 2019, with 8,548 procedures performed in 2020 (92). The incidence of GAS utilization among transgender people is mainly influenced by age, income, and race, with the primary variable for GAS utilization being income (90).

It should be noted that, while gender affirming surgeries are commonly associated with transgender patients, many of these procedures are similarly implemented in cis patients. For instance, a cisgender woman undergoing a breast augmentation may do so for gender affirming purposes, and cis men who have had genital damage may seek cosmetic surgical changes as a form of gender affirmation (93, 94). Individuals born with genital differences may also undergo gender affirming procedures (95).

### Future directions

#### A change in policy

Although gender-affirming medical (hormonal) therapy and surgical treatments are associated with a significant improvement in gender dysphoria (91, 96–98), there remains a paucity of data measuring outcomes across the many treatment domains, including complications and physical and mental health outcomes. Section 1557 of the Affordable Care Act became effective July 18, 2016, prohibiting discrimination in healthcare on the basis of a number of protected characteristics, including sex. The new regulations codified existing informal guidance from the Department of Health and Human Services, interpreting this provision to include protections from discrimination based on gender identity and sex stereotyping. Section 1557 regulations explicitly prohibit discrimination based on gender identity—making it clear that most insurers cannot deny or limit coverage because a treatment is related to an individual's gender transition. This change in codification, which increased access to medical and surgical therapy relevant to transgender individuals, resulted in a rise in the number of gender affirming surgeries performed in the US (90), as well as the number of gender health programs in this country.

### Pain phenotypes in patients undergoing gender affirmation surgeries

Despite the increasing incidence of gender affirming surgery (GAS), few studies have analyzed the prevalence and severity of post-operative pain immediately following GAS. Longer term reviews are sparse and yield conflicting results. To date, there are few reviews summarizing quality-of-life outcomes following gender-affirming surgeries over periods of greater than 1 year (99–101). Of the data that does exist, two long term follow up studies, found that gender affirming surgery alleviates gender dysphoria by improving quality of life, and psychological symptoms but also that there are still high psychiatric morbidity and suicide rates after gender reassignment surgery compared to the general population (98, 102). Several informative reviews focus on specific types of surgery, including phalloplasty (103, 104), vaginoplasty (105, 106), and laryngoplasty (107, 108). Furthermore, a recent review examined outcomes of chest, facial, and vocal cord surgeries (101). The consensus across reviews is that surgery improves transgender individuals’ quality of life. These reviews, while informative, do not explicitly consider longer term (i.e., > 1 year) quality-of-life outcomes following gender affirming surgery, or compare outcomes for each type of gender affirming surgery in transgender men and women. Patients tend to report positive transitioning outcomes within the first year after surgery (101, 109, 110). In a longer-term evaluation of transgender women's health and well-being after feminizing surgery, Lindqvist (111) found that transgender women reported increased health-related quality of life after feminizing surgery, but health-related quality of life declined in the following years. Hence, early evaluations (i.e., < 1 year), while informative, need to be supplemented with information about longer term functioning.

There is limited research on persistent postsurgical pain following mastectomy for gender dysphoria, even though it is well documented and common after mastectomy for breast cancer (112). In oncologic mastectomies, the likelihood of developing chronic pain is commonly appreciated and is often associated with an enduring neuropathic phenotype (113, 114). When breast tissue is surgically removed, sensory nerves traveling through these tissues are transected, stretched, or are caught up in scar during the healing process. These nerve injuries can lead to chronic pain due to the development of neuromas and/or scar contraction. Altered sensation, including “pins and needles”, shock-like, burning, or stabbing pain provide evidence of nerve injury as a cause for the pain. Response to local anesthetic nerve blocks can confirm that the source of the chronic pain is the injured nerves. The degree and frequency of persistent pain has been linked to the intensity of acute postoperative pain (115), pre-existing chronic pain conditions (116), as well as psychological factors such as anxiety and depression (117). Using acute postoperative pain scales and opioid consumption, Verdecchia and colleagues (118) found that the transgender top surgery patients consumed fewer opioids and reported lower pain scores than cisgender breast reduction patients. This may be explained by the effects of prior ongoing gender affirming hormone therapy (GAHT) on pain scores but more research is needed to confirm this (118). Similarly, Robinson et al. (119) prospectively compared top surgery to oncologic mastectomy without reconstruction and to mammoplasty breast reduction. They found that patients who underwent oncologic mastectomy consumed similar quantities of opioids to top surgery patients. In both studies, patients received pectoralis nerve blocks. A retrospective chart review (120) evaluated complication rates following top surgery, and surveyed patients about their experience with persistent postoperative pain. More than 25% of top surgery patients reported chronic pain, but 95% (*n* = 77) of these patients did not require analgesics. The potential role of concurrent hormone therapies in these studies was unfortunately not indicated. Generally, all participants were overprescribed opioids in the study. This may affect patients with a higher potential for substance abuse, which may be the reason these results conflict with Verdecchia and colleagues’ findings. The limited and conflicting data describing the prevalence and severity of pain following top surgery makes it challenging for physicians to counsel their patients on their risk of developing postoperative pain, as well as to predict opioid prescription needs.

Robust outcomes data after vaginoplasty and the prevalence of long-term gynecologic issues is lacking (121, 122). A past study that examined the prevalence of patient-reported symptoms and adverse reactions in 80 patients undergoing vaginoplasty found that 54% sought care within the first year after the procedure. The most common adverse outcomes included impaired wound healing / hypergranulation (13% / 39%), urinary dysfunction (19%). Sexual dysfunction issues were noted (34%) with anorgasmia (11%) and dyspareunia (11%) being most frequent (123). In a systematic review and meta-analysis that examined the outcome and complications of 3388 transgender females after penile inversion vaginoplasty, the mean prevalence of reported urinary complications ranged from 5.0%–11.9% with the most common symptoms being splayed stream (11.7%), meatal stenosis (6.9%), and irritative symptoms (frequency, urgency, nocturia) (11.5%) (124). In trans men, phalloplasty and metoidioplasty are the two most common genital surgeries (81, 125). Metoidioplasty is reported to have a complication rate of 10%–37% with postvoid dribble and/or a spraying stream (33%) and urethrocutaneous fistulas (5%–23%) being the most common (81). Phalloplasty may employ a free flap procedure, often with the radial forearm providing the donor site and the target tissue innervation. Most post operative reports on bottom surgery focus on the recovery of sensory function in the genitalia whereas with breast surgery a neuropathic phenotype has been readily reported. Such neuropathic phenotype reports are virtually nonexistent following vaginoplasty and penile construction. procedures. Carlotta et al. concluded that recovery of bottom sensation post-surgery suggests a greater sensory reinnervation, both in magnitude and temporally of the genitalia than that observed in the regeneration of limb afferents (126).

Hormone therapy can influence pain in transgender patients, with testosterone therapy improving chronic pain in some transgender men, for example (55). Furthermore, mood disorders such as anxiety can help to predict persistent post-mastectomy pain syndrome in women after oncologic mastectomy (127), yet similar work has not been described in transgender individuals. Local nerve blocks could be beneficial for pain management and deserve further systematic study, as they have been demonstrated to improve pain outcomes following oncologic mastectomy (128, 129).

### Future directions

Current insights into the characteristics of the pain phenotype occurring following gender affirmation surgical procedures suffer from: (i) limited attention to definitive descriptions and characterizations of the pain being reported by the individual, (ii) absence of long term follow up and appropriate and adequate characterization of the techniques employed, and (iii) influence of patients’ hormonal status on pain. Slow recognition of the pain state following breast intervention in cis women makes it unlikely that pain states in trans people will be evident unless they are specifically characterized.

There appears to be a relative lack of persistence of pain and ongoing pain phenotypes after gender affirming bottom surgery. This stands in contrast to the time course of postoperative pain for other surgeries of skin and bone, where significant pain may persist and develop into a neuropathic phenotype. The rapid reduction of pain that occurs in the bottom surgery patient may be intertwined with relief of gender dysphoria and merits further research (126).

Further, additional work characterizing anatomy and neuroplasticity is essential for understanding the nature of pain in transgender individuals. Such understanding is not only important for clinical management and determination of prognosis but is essential as a part of supporting the patient by giving an explanation for their symptoms, both pain and otherwise. Finally, it should be clear that predictions regarding the pain state in transgender patients that are based solely on those of the cis individual (as in mastectomy and breast augmentation, or genital reconstruction/bottom surgery) do not include the potential impact of transgender hormonal therapeutics or the mental health impact of gender dysphoria, societal marginalization, and social gender transition. Finally, as noted in the following section, hormonal therapies are an important component of the treatment regimens of the transgender individual and the effects of such treatment on the post operative pain state has been poorly considered, and future work in this would importantly consider this interaction.

## Hormone replacement therapy (HRT) in adults

Hormone replacement therapy is a cornerstone of gender affirming medical care to address gender dysphoria (62, 73, 130). The National Transgender Discrimination Survey on Health and Health Care reports gender-affirming hormone therapy (GAHT) in up to 80% of transgender people. Other estimates suggest that approximately 30%–68% of transgender persons in the US utilize hormone therapy in pursual of congruent secondary sex characteristics (36, 131–133). In gender-diverse people assigned female at birth, GAHT primarily involves administration of exogenous testosterone esters (130, 134) for masculinization. Many transgender men use testosterone therapy to suppress estrogen (134). In transgender women, hormone therapy includes use of a synthetic estrogen, usually with anti-androgens to suppress testosterone (135), and occasionally progesterone, though the risk/benefit ratio of progesterone use in trans women is understudied (136). It is important to note that the trans population is heterogeneous in therapeutic hormone use, and includes different formulations dosages, and routes of administration (oral, injection, patches). For example, while the clinical goal for some trans women may be to maintain serum concentrations of estradiol similar to those of cis women (100–200 pg/ml) (130), other patients may seek the phenotypic and physiologic effects of lower concentrations. Alternatively, trans people might have only sporadic access to prescribed therapy or may obtain and utilize hormone therapy outside of medical direction.

### Hormone therapeutics and pain in transgender individuals

Evidence describing the effect of hormone replacement therapy on the experience of pain in transgender populations is inconsistent and is often limited by small sample size and subject to sampling bias. One exploratory study of trans men in Thailand found that those undergoing testosterone therapy reported higher bodily pain scores on the Short Form Health Survey 36 compared with those not utilizing testosterone (137). Conversely, a questionnaire-based study of 26 trans men found that 60% reported a significant improvement in chronic headache symptoms that had been present prior to the start of testosterone treatment (55). Meanwhile, a study of Israeli trans men did not find a significant difference between the prevalence of fibromyalgia symptoms before and after the initiation of hormonal therapy (53). A U.S. survey-based study of 100 trans men who had undergone phalloplasty and who were taking testosterone found that 51% (48/94) reported pain with sexual penetration. Pain was present before initiation of testosterone in 41.7% (20 of 48), and no significant correlation was found between pain scores and the duration of time since initiating testosterone (60). In another survey-based study of 183 trans men endorsing abdominopelvic pain, 69.4% (127/183) reported new-onset pain following initiation of testosterone GAHT, with a median time from testosterone initiation to pain onset being 1 year (138). In summary, insufficient data exist to definitively discern the specific effects of testosterone GAHT on pain in trans men.

In contrast to the small number and heterogeneity of findings observed in studies describing pain in trans male individuals, the data describing trends in pain among trans women are more consistent. Several studies have suggested that trans women experience more pain than cis men and cis women, a phenomenon that appears to be exacerbated by anti-androgen therapy and an estrogen GAHT (54, 56, 138–140). In a study of 47 trans women, chronic pain was observed in 29.8%, with breast pain and headache onset occurring after initiation of estradiol and/or anti-androgen therapy in 11 of 14 affected individuals (55). Further, among trans women taking anti-androgens and high-dose estrogens, the prevalence of migraine has been suggested to increase, particularly among those taking high doses of oral estrogens (55). A positive correlation has also been demonstrated between trans women's time on GAHT and their experience of bodily pain (54). Overall, the prevalence and severity of pain in transgender women is associated with impaired mental health outcomes, with Female Sexual Function Index Pain scores (e.g., pain associated with sexual activity) accounting for a significant proportion of the Beck Depression Inventory depression score, after controlling for age and general mental health levels (139).

Research investigating pain in transgender children and adolescents is very limited. While injection site pain is a well-known side effect of any injectable medication, it is unclear whether gonadotropin releasing hormone (GnRH) agonists that some gender-diverse youth use for pubertal suppression are associated with altered pain experience. During initial cyclical administration of GnRH agonist therapy, the pituitary is stimulated to produce gonadotropic hormones which increase downstream gonadal steroid secretion. Infusions of GnRH agonists produce an initial transient increase in sex hormones, but with continued non-pulsatile stimulation, LH and FSH synthesis are inhibited and estrogen and testosterone levels decline (141). Of note, GnRH agonists have been used as a last line of pain suppression in adolescent cis girls suffering from dysmenorrhea, endometriosis, and chronic pelvic pain. However, their study did not include gender diverse patients (142–144).

### Hormone replacement and pain in adults

Here we briefly review the two primary classes of agents used in GAHT and their effects on pain processing. We first examine the current literature on the effects of the gonadal steroid hormones on pain in animals and cis gendered men and women to hypothesize their effects on pain in transgender individuals.

#### Testosterone & pain

Regarding androgenic influence on pain, one study collected pain scores of 127 cis gender men and 145 cis gender women. The study reported that testosterone levels were negatively correlated with pain after knee replacement at the operated knee, e.g., high testosterone was correlated with lower pain scores, in both cisgender men and women osteoarthritis patients (145). Similarly, androgen level has been reported in limited reports to be negatively correlated with pain in a study of 40 cycling women tested with noxious stimuli in the lab (146), as well as negatively correlated with days/month of pelvic pain, menstrual pain, and headache in 56 cycling women (147). In laboratory rodents, exogenous testosterone administered to adults may increase pain thresholds (i.e., decrease or delay response to noxious stimuli) in gonadectomized males and females [e.g., (148)], although testosterone administered to gonadally intact rodents may not alter pain in either sex [e.g., (149)]. Because testosterone (or estradiol) administration to gonadally intact animals triggers negative feedback to the hypothalamic-pituitary-gonadal axis (thereby decreasing endogenous gonadal hormone release) (150), exogenous hormone effects may differ in gonadally intact vs. gonadectomized individuals. When androgens do reduce pain, the reduction coincides with its attenuating effects on inflammation (151). However, it has been shown that pelvic pain in transgender men often occurs after initiation of testosterone therapy (152). Given the known androgen sensitivity of the pelvic floor musculature (153), further research into pelvic floor muscle dysfunction is warranted.

#### Estrogens & Pain

Menstrual cycle phase may alter pain in cisgender women with migraine, fibromyalgia, and temporomandibular pain; typically, pain worsens during periods of rapid estrogenic decline, such as during the late luteal phase (154). However, there is disagreement among studies examining the interactions between ovarian hormones and pain (155). In women without chronic pain, somatosensory thresholds are not systematically affected by cyclic changes in ovarian hormones (154, 156). In laboratory rodents, estradiol may increase pain-related behaviors in both sexes (157), but estradiol has also been observed to decrease pain-related behaviors (e.g., in gonadally intact females using a visceral pain model (149). It has been suggested that estrogenic influence on pain depends on the extent to which a given pain disorder involves immune, skeletal, cardiovascular (and other) systems, because estrogens can influence each of these systems differently (158). Estrogens have diverse effects on peripheral organs and tissues, and in the brain as mediated by estrogen receptor alpha (ERalpha, playing a critical role in reproductive neuroendocrine function and behavior) and estrogen receptor beta (ERbeta, present in populations of hypothalamic GnRH, corticotropin releasing hormone, vasopressin, oxytocin, and prolactin expressing neurons) (159).

Estrogenic modulation of pain may depend on the type of chronic pain (or pain model in animal studies), whether the estrogen-treated individual is gonadectomized, and, among other variables, whether estrogens are combined with progestins (157, 159).

#### Other GAHT therapeutics

To support gender transition, anti-androgenic treatments are sometimes used; these include 5α-reductase inhibitors, progestins, and the mineralocorticoid receptor/androgen receptor antagonist spironolactone which functions as a testosterone blocker (160). Little is known about the impact of these drugs alone or in combination with estrogens—which is how they would typically be taken for gender transition—on chronic pain. Spironolactone in isolation has been reported to decrease inflammatory pain-related behavior in male mice (161) and neuropathic pain-related behavior in rats (162), but it did not alter fibromyalgia pain in women (163), or pain in human osteoarthritis patients of either sex (164). Research is needed to determine whether combined estrogens + anti-androgens are likely to affect chronic pain in preoperative vs. postoperative trans individuals.

### Future directions

It should be stressed that there is a relative paucity of data substantiating the relationships between hormones and pain expression in transgender and cisgender individuals. Additional work in this area promises insight into effective pain management for people, especially those receiving exogenous hormones.

### Mechanisms driving gonadal hormone effects on pain

Aside from their effects on inflammatory cascades, steroid hormones appear to modulate cortical processing of pain-related stimuli (165–167), and preclinical studies using rodents have shown differential excitability of CNS targets in male and female organisms by the same endogenous steroid hormones (168). Although steroid hormone action predominantly involves nuclear signaling and regulation of gene expression, gonadal hormones have also been shown to exert direct influence on the peripheral and central nervous systems (158, 168–173). Mechanisms potentially underlying these phenomena include pronociceptive interactions between the G-protein coupled estrogen receptor, the ionotropic 5HT3A serotonin receptor, NMDA receptors, and glutaminergic signaling in the CNS (174–176). Estrogens may also negatively regulate inhibitory neurotransmission through glycine and GABA downregulation (177–179). Importantly, estradiol may also negatively regulate the antinociceptive endocannabinoid and opioid systems (180–182). Together, these mechanistic data suggest a pronociceptive effect of estrogens that may contribute to the higher levels and rates of pain in cisgender and transgender women observed in cohort and population-based studies. However, it should be noted that these studies have been conducted in cisgender individuals, and their findings may not be generalizable to transgender and gender-diverse individuals.

### Hormone modulation of analgesic drug action

Gonadal hormones can influence the potency/efficacy of some analgesics and anesthetics. The extent of this influence among transgender individuals who take exogenous hormones may also depend on whether they stop hormones before surgery. In fact, the practice of stopping hormones is losing popularity as the literature grows with evidence that hormone cessation is not necessary. For example, in male and female rats, testosterone can increase morphine's antinociceptive potency on some but not all tests of acute pain (183). Estradiol also modulates morphine antinociception in gonadectomized female rats, but the direction of effect depends on estradiol dose, site of administration (peripheral or central), and timing (184); these shifts are paralleled by modest fluctuations in morphine potency across the estrous cycle (185) but see (186).

Finally, the testosterone blocker spironolactone, commonly used in the U.S., has been found to increase the antinociceptive effects of morphine and oxycodone in male rats, and this interaction appears to be pharmacokinetic (187, 188). Centrally, the antinociceptive effects of morphine are mediated, in part, via binding to the mu opioid receptor (MOR) within the midbrain periaqueductal gray (PAG). Both androgen (AR) and estrogen (ERα) receptors are located within the PAG, making this a likely anatomical substrate whereby gonadal steroids influence pain and analgesia (189). Several mechanisms have been proposed to account for gonadal hormone modulation of opioid analgesia. First, estrogens have been shown to attenuate MOR signaling by uncoupling MOR from its K + channel, thereby reducing morphine-induced hyperpolarization (179, 190), and by inducing MOR internalization resulting in reduced receptor availability (191). Estradiol has also been shown to facilitate the formation of MOR/KOR heterodimers in the spinal cord, thereby augmenting morphine's effects in females (192). Neuroimmune factors have also been implicated. In rodents, low doses of estradiol, comparable to normal circulating levels, increase proinflammatory cytokine production (193), which is known to reduce morphine antinociception in a TLR4-dependent manner (194–196). Opioids are metabolized via glucuronidation to produce two metabolites: morphine-6-glucuronide (M6G), which has a high affinity for MOR and is considered pro-analgesic, and morphine-3-glucuronide (M3G), which has high affinity for TLR4 and opposes morphine action. In rodents, M3G levels are 2-fold higher in both plasma and PAG following systemic morphine (197), which is likely why direct PAG administration of the MOR-selective metabolite M6G results in a greater analgesic response than morphine alone in females (195). Opioid and barbiturate pharmacokinetics may be altered by both androgens and estrogens as shown in rat models [e.g., (198–201)]. Overall, a multitude of pharmacokinetic and pharmacodynamic mechanisms underlie sex chromosome and gonadal hormone modulation of analgesia and anesthesia, some of which may be relevant to transgender individuals who have chronic pain or undergo surgery.

### Future directions

The preclinical model does not presume to mirror the complexity of the estradiol and testosterone modulation of pain in transgendered populations. However, the preclinical models can offer direction for future studies to be observed in transgender populations. Although there is a growing literature, at least in laboratory animals, examining estradiol modulation of pain in rodents identified as female at birth and testosterone modulation of pain in rodents assigned male, there are few preclinical studies on hormone reversal using gonadectomized rodents and almost none in gonadally intact rodents. Both types of studies, but particularly the latter, are needed to model the biological effects of hormone treatment alone, or hormone treatment with orchiectomy or oophorectomy, in transgender patients. Additionally, in trans women, the use of estrogenic treatment is often accompanied by an anti-androgenic treatment (i.e., testosterone blockers), but very little is known about the combination of estrogens plus commonly used demasculinizing treatments on chronic pain in either clinical or preclinical studies. Studies manipulating gonadal hormones are relatively straightforward to conduct in laboratory rodents. Combining these manipulations with a variety of chronic pain models that have predictive validity (35) will greatly improve our understanding of how interventions common to the human gender transition process may affect pain associated with various pathologies. Additionally, gonadal hormone manipulations in rodents that mirror those used for human gender transitions could be used to further our understanding of how gonadal hormones are likely to affect the potency and efficacy of analgesics and anesthetics commonly used to treat pain clinically, including in transgender individuals.

A second potentially important issue relates to age or developmental stage of hormone exposure (as discussed above in puberty suppressive interventions). We do not know whether age influences the impact of hormones on pain and analgesia. This question could be readily addressed via laboratory rodent studies that examine the impact of hormone manipulation (via exogenous hormone treatment with and without gonadectomy) at pre-pubertal vs. post-pubertal ages (and for the latter, from adolescent to older adult) on pain and analgesic potency/efficacy. Nearly all pain research in rodents is currently conducted in post-pubertal adolescents to young adults (37). Because individuals may seek medical interventions for gender transition at various ages, it is imperative to determine whether the impact of typical hormone treatment regimens on pain and analgesia is age-dependent. To the extent that sex differences in anesthetic effects are known to change with age (202, 203), it might be predicted that the impact of gender affirming hormone treatments on pain and analgesia will depend on an individual's age. These observations in preclinical models are clearly suggestive of the information and clinical research issues that remain to be defined in transgender humans.

### Hormone therapeutics for pubertal suppression in transgender youth

An important focus of hormonal therapeutics is the use of gonadotropin-releasing hormone analogues used to suppress endogenous puberty in transgender adolescents. The incidence of such therapy was reported to be 2.5% (∼515) of transgender people (*n* = 20,619) in one study (204). In select cases, both cisgender and transgender youth both utilize pubertal inhibition (“puberty blocker”) therapy. It should be noted that this therapeutic intervention is not new but has been utilized since the 1950s for use in children with central precocious puberty which results from premature activation of the hypothalamic-pituitary-gonadal (HPG) axis, and for endometriosis. Because of this, use in puberty suppression is explicitly an on-label use for GnRH analogues (205–207).

Gonadotropin-releasing hormone (GnRH) agonists delivered as slow-release depot formulations are prescribed to suppress puberty for transgender adolescents. GnRH agonist therapy inhibits gonadal sex steroid production by persistently activating and desensitizing the GnRH receptor. This desensitization suppresses luteinizing hormone and follicle-stimulating hormone release from the anterior pituitary gland preventing the progression of puberty for the duration of GnRH agonist use (208). GnRH agonist use has been a component of therapy to manage gender dysphoria in adolescents (209). However, because GAHT is restricted to those above age 16 (recent guidelines suggest potential for use as early as 14), pubertal inhibition therapy is provided following reaching Tanner stage 2 as an intervening therapy. This provides the individual with more time to decide what physical interventions they may prefer. Several studies and reviews have suggested favorable mental health outcomes of puberty blockade (210–212). The basis for these outcomes is that pubertal suppression prevents gender-incongruent changes which are not easily reversible (e.g., breast development in trans boys, changes in voice and bone structure in trans girls) (204, 213–215). Beyond mental health, liabilities of pubertal suppression are poorly studied and controversial. Preclinical data has shown GnRH to have multiple effects outside of the hypothalamic-pituitary-gonadal (HPG) axis (216). While pubertal suppression with long term use of GnRH agonist therapy has been shown to have a positive effect on issues such as gender dysphoria, a variety of long-term effects have been identified, including polycystic ovary syndrome in trans boys, changes in body composition, metabolic profiles and bone mineral density, along with short term side effects such as headaches, hot flushes, mood swings and injection site reactions (rashes, bruising and sterile abscess formation) (217). Aside from the sequelae of acute treatment, systematic assessments data of the long-term effect of puberty suppression and pain biology is needed (218).

Research investigating pain in transgender children and adolescents on pubertal blockers is limited. While injection site pain is a well-known side effect of any injectable medication, it is unclear whether GnRH agonists used for pubertal suppression are associated with altered pain experiences in gender diverse youth. Adults and post-pubertal adolescents may therefore experience breast, pelvic, or testicular pain during the initial steroid hormone administration within the first 3 months, while recurring administration to achieve gonadal suppression may be associated with a moderate incidence (<10%–20%) of arthralgias, headaches, anxiety/depression/ irritability or dyspareunia among individuals with vaginas (143, 144). In addition, as GnRH agonists are first line therapy for the treatment of endometriosis, initiation of this therapy may lessen endometriosis-related pelvic pain, dyspareunia, and dyschezia in trans men (143). Despite the complex interactions between GnRH agonists and other forms of gender affirming hormone therapy, a retrospective case series of 8 youth (6 AFAB, 2 AMAB) diagnosed with gender dysphoria during chronic pain treatment demonstrated improved scores on the Pain Burden Interview, Functional Disability Inventory, and Pain Catastrophizing Scales which coincided with synergistic therapies focused on gender affirmation and pain (72). However, a study that compared 4,778 transgender teens to 630,200 cisgender students in California middle and high schools found on a self-report survey that transgender students were about 2−1/2 times more likely than non-transgender students to have used cocaine/methamphetamine and about 2.8 times as likely to have experienced use of inhalant, twice as likely to have used prescription pain medication and more than 3 times as likely to use cigarettes (219). Additional research that examines possible correlations among endogenous hormone suppression, exogenous hormone administration, mental health, gender dysphoria, and pain in transgender populations is needed. Further, is this important to consider in relationship to other variables, such as surgical status, more complex regimens (including progesterone, for instance), previous use of GnRH therapy, etc.? What might these studies look like? What kind of patient profiles should be detected? What about in cis women with low estrogen or cis men with high estrogen?

### Future directions

While puberty suppression is effective in managing the gender dysphoria for many young transgender patients, there is little work characterizing the long-term impact of this treatment on pain or analgesia. The preclinical data reviewed here makes the case that this issue should be studied in more depth due to this treatment being used as a main suppressor of puberty. Further, will remain important this important to consider. Pain expression and phenotype in relationship to other variables, such as surgical status, more complex regimens, previous use of GnRH therapy, etc.? What might these studies look like? What kind of patient profiles should be detected? What about in cis women with low estrogen or cis men with high estrogen?

## Sex differences in immune/inflammatory processes

Considering the changes in patient biology that result from gender affirming medical procedures in many transgender individuals, it is worthwhile to briefly consider sexually dimorphic responses to pathogens, immunizations, and inflammation (66, 220). As examples, dendritic cells isolated from cis women are generally associated with increased Type 1 interferon responses upon activation, as compared to dendritic cells from men, while stimulated macrophages/monocytes in cis women generally release significantly more pro-inflammatory cytokines (220). These divergent inflammatory responses contribute, in part, to the sex-specific differences observed in HIV-1 (221) and SARS-CoV-2 (222, 223) pathologies, and they have been attributed to (i) differences in gonadal hormones and ii) genes located on sex chromosomes. Social factors also contribute to one's risk of infection, and gender has been shown to play a role in behaviors associated with contracting COVID-19, such as wearing masks.

Indeed, in addition to peripheral immune cells, estrogens can influence inflammatory responses of CNS microglial cells and astrocytes. It is hypothesized that estrogens can exert a neuroprotective, anti-inflammatory effect in Parkinson's disease, by attenuating microglial and astrocyte responses to inflammatory stimuli (224). In addition to observational studies linking estrogens with reduced Parkinson's risk, estrogens have been used as a treatment for Parkinson's disease symptoms (224, 225). A similar mechanism is hypothesized for the apparent effect of estrogen treatment to reduce neuropathic pain (226, 227). That is, estrogen reduces microglial and astrocyte inflammatory responses via activation of estrogen receptors within these cells (226–229).

### Hormone replacement and immunity

Membrane-associated estrogen receptors (ESRs) act as G protein-coupled receptors to rapidly alter gene regulation and protein function (132, 230). Intracellular ESRs act as hormone-dependent transcription factors. Thus, the presence or absence of systemic estrogens directly influences immune cell transcriptome and functional activity. Not surprisingly, medically guided increases or decreases in circulating estrogens are highly relevant to immunity and inflammation, as nearly every cellular subset of the adaptive and the innate immune system express estrogen receptors (ESRalpha /ESRbeta (231). The influence of estrogens on immune cell function has been widely observed (220), with estrogen associated with reduced cytotoxic functional capacity of innate Natural Killer (NK) cells in animal models (232), e.g., lymphocytes that bind and kill tumor and virus-infected cells without antigen stimulation. In humans, not only do circulating NK cells isolated from cisgender women demonstrate reduced killing compared to those isolated from cisgender men (233), but cytotoxic capacity of NK cells isolated from those who take oral contraceptives is lower than those who do not (233). Furthermore, NK activity increases during menopause (234), when estrogen levels are reduced, and decreases during pregnancy (235), when estrogen levels increase. This ability of gonadal hormones to directly modulate NK function could be highly relevant within the context of neuropathic pain in transgender persons utilizing GAHT (236, 237).

The gonadal hormone milieu in which immune cells mature also influences their subsequent responsivity to estrogen. Latency reversal agents can be used *in vitro* to induce CD4 + memory T cells silently infected with HIV to produce viral RNA (238). With cells isolated from HIV + cis men (cells that presumably matured in a relatively “low estrogen” environment), the potency of latency reversal agents is reduced by simultaneous treatment with an estrogen. In contrast, in cells that matured in cis women, a relatively “high estrogen” environment, this reversal was completely abrogated by co-treatment with an estrogen. The duration and consistency of GAHT in trans persons could therefore result in a mosaic of gonadal hormone-responsive immune cells. Relatively short-lived innate immune cells might take on the associated transcriptome and phenotype of maturing in their therapeutic environment relatively quickly, similar to what is observed in post-menopausal cis women who utilize hormone replacement therapy (234). Conversely, long-lived adaptive and tissue-resident immune cells might retain the phenotypic characteristics (e.g., hormone responsivity and receptor expression) of the sex assigned at birth for more extended periods. Central nervous system microglial cells, for example, can persist for decades (239).

### Future directions

Given the influence of gonadal hormones on immunity and inflammation, ensuring that transgender populations are included in basic, translational, and clinical research trials is important. Transgender persons who choose gender affirming therapies could have immune and inflammatory profiles distinct from those of cisgender individuals. Duration and consistency of GAHT should be considered, and study entry criteria that excludes transgender individuals from participation should be explicitly acknowledged. Thus, while the influence of gonadal hormones on peripheral and CNS inflammation and immunity has been investigated in a sex-binary manner, similar studies examining gonadal hormones in transgender persons are necessary (240).

### Sex chromosomes

Consideration of the issues of pain patients (241, 242) requires consideration of chromosomal status. Sexual differentiation of pain sensitivity may begin very early in life in humans (243) and in preclinical models (244). As discussed below, preclinical studies show that chromosomal and gonadal hormonal (organizational and activational) mechanisms robustly contribute to sexual differentiation in the expression of the pain phenotype and correspondingly interventions that down regulate the transmission of the afferent signal and its integration, [e.g., analgesia (183, 245, 246)].

Healthy adult cis women typically show lower pain thresholds and pain tolerance than men when tested with various noxious stimuli (5, 242, 247), and when such evoked thresholds were assessed in patients with mononeuropathies (248). As discussed below binary differences have been typically reported in preclinical models. In migraine, inflammatory phenotypes such as rheumatoid arthritis, temporomandibular disorder, and conditions of widespread pain such as fibromyalgia are likely due to the role played by circulating immune complexes acting though adaptive immune signaling. The incidence of these pain conditions may be up to 2–3 times more common in women than men (40, 241, 249–251). As a caveat, one must consider that as discussed above, there is a confound in the sex dependent differences given the influences of sexism and other social factors where providers have been shown to dismiss, under-medicate, and underreport women's pain.

These observations are consistent with the appreciation that the variation in human immune and inflammatory responses is robustly influenced by sex chromosomes (252, 253). Understanding the contribution of sex chromosomes to the pain phenotype complicated by their organization. The sex chromosomes are routinely considered in terms of their common pairing as “XX” or “XY”. In fact, a multitude of potential combinations exist, and often the number of X chromosomes directly influences inflammation and immunity (254, 255). While the X chromosome is not necessarily enriched for immune genes as compared to autosomal chromosomes, it encodes for a number of highly relevant genes, including genes associated with the inflammatory transcription regulator, nuclear factor B (NF-kB) (255, 256). In addition, mutations within these essential genes can result in severe immune defects in individuals with only one X chromosome, as for example in X-linked severe combined immunodeficiency (XSCID) (257). Regardless of the number of X chromosomes present, additional X chromosomes are typically silenced epigenetically (253, 258–260). In the absence of mutations, the presence of more than one X chromosome is broadly relevant to the immune and inflammatory phenotype (258). Defining the impact of these different X pairings has been a subject of interest in preclinical literature and will be discussed further below.

### Future directions

The interactions of chromosomal constituency and GAHT must be defined scientifically in transgender populations. Consideration of both gonadal hormones and sex chromosomes, e.g., “estrogen rich XY” or “estrogen low XX”, will be of scientific and practical importance in further elucidating sex- and gender-specific pathways of inflammation and pain. It is important to note that a variety of complexities serve to complicate the role of chromosomes and pain. Thus, for example, what might be the contributions of non-46,XX/46,XY chromosome combinations? What is the role of the Y chromosome in activating a cascade or does “silencing” the second X chromosome?

## Environmental exposure: impacts of chronic social stress

Transgender persons frequently navigate a “pro-inflammatory” social environment which as reviewed at the outset have demonstrably significant effect upon morbidity and quality of life (261). Both acute and chronic exposure to social stressors, such as rejection, isolation, and actual or anticipated threats of physical assault have been shown to generate physiological inflammatory responses (262, 263). Social stress induces expression of NF-kB, leading to increased concentrations of circulating soluble markers of inflammation, such as IL-6 and TNF-alpha (264–267). While this is true for all individuals regardless of sex or gender, trans persons disproportionately experience the burden of (i) negative external stressors, (ii) the stress of hypervigilance in anticipation of negative external stressors, and (iii) socially conditioned and internalized stress (268).

Furthermore, the Gender Minority Stress and Resilience (GMSR) model revealed that these chronic social stressors encourage trans individuals to adopt negative coping behaviors (e.g., substance abuse) which in turn also negatively influence immunity, inflammation, and health outcomes (269).

It has therefore been hypothesized that chronic exposure to social threats is a primary driver of systemic inflammation and health disparities in sex- and gender-minority populations (263). A study of trans men utilizing GAHT, typically testosterone therapy to suppress estrogen, reported that serum C Reactive Protein (CRP) concentrations, a measure of inflammation, were associated strongly with the emotional stress related to being misgendered and misnamed during daily activities. Examination of diurnal cortisol functioning in transgender men undergoing testosterone therapy revealed that elevated diurnal cortisol levels at awakening were associated with transition-specific social stressors, such as those experienced during transitioning, coming out and common life events such as utilization of gender-specific public bathrooms (270). This is highly relevant to pain experiences in transgender persons, as elevated concentrations of circulating CRP have been associated with pain sensitivity (271); however, this topic is largely underexplored within the literature.

Reviews of available literature have attempted to extrapolate data associating stress in transgender men and women to migraine incidence. These reviews have concluded that social stress, particularly stress induced by stigma in an unsupportive healthcare environment, in combination with GAHT, may contributes to the higher rates of migraine in transgender persons (272). This represents a substantial gap in the primary research literature which should be addressed directly with research addressing migraine in cis and trans populations (273). For context, many classes of environmental and societal stressors have been shown to have a direct impact on a number of measured variables, including: psychological processing, concern over financial well-being such as access to affordable housing (274), general concern over health (275), access to health care (276), and food insecurity (277).

While a survey of over 2,000 trans and gender non-conforming participants found no differences in chronic health conditions or health behaviors (278), these data are of limited utility as the survey was phone-based, and thus excluded potential participants who have experienced more severe social stress (poverty, homelessness or incarceration). Furthermore, physiological effects of social stress can be mitigated by familial and social support (279). An exploratory study found no associations between gender minority stress and resilience scales and circulating concentrations of CRP in young trans persons receiving GAHT (279, 280). Notably, 84% of these older teenagers and young adult participants lived with their parents, indicative of stable familial support, perhaps suggesting that a supportive familial structure is “anti-inflammatory” in an otherwise “pro-inflammatory” society (281, 282). These observational studies highlight the importance of family support for psychological wellbeing as well as for material survival (adequate health insurance might only be possible through parents or spouses, financial security through social security income relies on traditional family structures). This is a challenge for many transgender persons, many of whom lose their family support structure when they transition (283).

### Future directions

When examining inflammation and pain outcomes in transgender populations, recruitment strategies, data analysis, and conclusions should consider the pro-inflammatory influence of social stress. Studies inclusive of transgender populations in the United States could be dominated by recruitment within urban areas, potentially overlooking the diversity of rural America. The Movement Advancement Project (MAP) released a report in 2019, that included an original analysis of the unique challenges and opportunities for transgender people in rural America. Although discrimination occurs as frequently in rural and urban areas, the structural differences in rural America amplified the effects of discrimination due to less employment opportunities and healthcare options (284). For future studies addressing the impact of social stress on transgender health, it will be important to consider the diversity of different geographic areas globally and to include both urban as well as rural populations. Recruitment strategies (e.g., telephones, engagement with advertisements on social media) could inadvertently exclude transgender persons who have experienced or are currently experiencing severe social stress. Recruitment, research, and clinical staff who interact with transgender participants should receive appropriate training to eliminate introduction of known pro-inflammatory social stressors into the research process (e.g., mis-gendering, mis-naming, and judgmental or exclusionary comments) and should preferably contain transgender team members. Data analysis and study conclusions must thoughtfully incorporate quantitative measures of participant stress. Transgender identity is not pathological, nor is it inherently a pro-inflammatory condition. However, stressors that trans persons disproportionally experience are typically considered to be pro-inflammatory, as indicated by endpoints such as CRP, as noted above (270, 280).

## Impact of emotion and body image on pain in transgender people

The report of a pain state results from both peripheral nociceptive signals and sensitization (e.g., tissue injury, joint inflammation) and effects of attentional and emotional elaboration in the brain (285). Attention heightens the perceived intensity of pain (286), while negative emotions increase its unpleasantness (287–289). Trauma, depression, and mood (290) alter the brain's emotional learning circuitry (291) and contribute to pain chronification (292–294).

As reviewed in the preceding section, trans people face disproportionate rates of violence (295, 296) and discrimination, including in healthcare (297). Violence and discrimination have significant biological consequences which are manifested in heightened negative affect and psychopathology. The gender minority stress model posits both external and internal stressors related to gender minority status (272), leading to higher prevalence of depression, anxiety, and suicidal ideation among transgender individuals relative to the general population (298–300). Prevalence of syndromes such as fibromyalgia, reflecting a likely role for circulating immune complexes resulting in amplification of pain processing, appears to be greatly heightened in transgender men and women (53). Violence is a certain risk factor for post-traumatic stress disorder (PTSD) (301), which in turn increases the risk for opioid use disorder (302). Anti-transgender bias and non-affirmation, which increase severity of PTSD symptoms (303) and risk of opioid misuse, highlight the need for systemic improvements in transgender inclusivity in medical and behavioral healthcare (304) and in peer support (298).

Gender dysphoria and body dysphoria constitute additional internal risk factors for heightened experiences of pain. Many transgender individuals experience significant gender-related body dysphoria and avoidance, particularly for body parts associated with sex differences (73, 292, 305, 306). For trans men, body dysphoria is often focused on breasts, genitals, and lack of facial hair (307–309), while in trans women, body dysphoria frequently focuses on the genitals, face, and presence of facial hair (310). The effect of body dysphoria and reduced bodily self-identification on the experience of pain in transgender individuals is not known. However, there is evidence that body satisfaction and body representation in general modulate experiences of pain. Thematic analyses of transgender persons’ descriptions of their body part-specific dysphoria have highlighted independent themes of disconnection from the body (feeling that a body part is viscerally wrong, deformed, or alien), and emotional distress (negative emotional reactions and hyper fixation) (311). Body avoidance and dissociation, which may be elevated in transgender patients (312), may reduce pain perception due to reduced attention to the body part, similar to patients with spatial (hemi-) neglect (192). There have been reports that modulation of pain perception can be induced by observing a rubber hand that was perceived to be the observers own hand (313, 314). In contrast, heightened negative affect surrounding a dysphoric body part may increase pain perception. In patients with arthritis, negative body image has been correlated with greater pain severity (315). Conversely, laboratory studies using rubber limb coverings mimicking an injured or hairy limb/hand appearance showed that negative body image increases acute pain (316).

Conversely, positive emotions such as gender euphoria may reduce experiences of pain. Transgender people appear to have lower rates of phantom breasts and penises after gender-affirming surgical procedures than cisgender individuals do after these body parts are removed for medical reasons (317, 318). In addition, transgender men exhibit lower pain and opioid consumption after breast removal than do women after mastectomy for cancer or risk of cancer; although hormone therapies may play a role, this difference may reflect congruence/gender euphoria of breast removal for trans men versus the distress and incongruence for cisgender women (as well as negative emotions associated with cancer) (118). An important caveat, however, is that these procedures are not identical for transgender and cisgender patients. Euphoria can also arise from phantom body parts aligned with gender and from the use of prostheses, increasing positive identification with the body (319, 320).

Concerns about bias or body dysphoria during clinical encounters may lead patients to delay seeking care for acute pain, elevating the risk of transition to chronic pain. Provider misgendering and microaggressions are likely to amplify gender dysphoria, leading to stress, and consequently, worsening or prolonging the pain.

### Future directions

We pose the following questions regarding the effects of body image on pain in transgender individuals: (1) What are the direct and indirect effects of gender and body satisfaction on pain in transgender individuals, and what variables affect this relationship? (2) Are certain pain conditions disproportionately experienced by transgender patients, and are they linked to dysphoric body parts, and/or to strategies of body avoidance or hyper fixation? (3) What is the impact of patient satisfaction with gender-affirming procedures on existing pain conditions or severity of postsurgical pain? (4) How can healthcare providers reduce body-related bias and microaggressions, and what is the effect of such improvements on body dysphoria and pain? (5) Are there novel approaches to improving body image in transgender individuals that might reduce acute or chronic pain?

## Neuroimaging in the study of gender identity and pain

A topic of enduring interest across many fields of neuroscience relates to the influence of sex and gender on human brain anatomy (321, 322), function, connectivity, and metabolism. Albeit the additive impact that gene expression, own-body metabolism, environment, and society can surely have on the behavioral differences documented between cisgender men and women (323), the question remains as to how putative anatomical, functional, and metabolic brain regional characteristics might also contribute—or be shaped by—gender identity and expression. It is of note that the traditional illustration of the body map in the brain, the homunculus, by Wilder Penfield refers to males with testicles, penis, and no breasts. Only 10 years ago there was a call for the need to a full mapping of the female brain with the production of a hermunculus as a first step toward fully understanding neurological and physiological sex differences related to pain conditions and pain management (324).

Gender and body dysphoria are associated with differences in neural body representation. Prior to any surgical procedures, some trans men have reported the sensation of having a penis (317, 319, 320, 325)—similar to the phantom limbs that frequently occur after amputation (326). This phenomenon may suggest a strong representation of gender-congruent body sex in the brain. Indeed, breasts that feel incongruent on trans men elicit a lower somatosensory response in brain areas associated with salience and body ownership, but increased activation in a brain region associated with alarm, in comparison to the activation imaging in cisgender people with congruent-feeling breasts (327). Correlated differences in brain connectivity suggest differences—whether innate or acquired—in body representation (327). Similarly, neuroimaging studies have reported that trans men show weaker connections between body perception and body ownership networks (328), and decreased connectivity within the default mode network (329, 330) and anterior cingulate cortex, the latter related to lower ratings of “self” for gendered body images (331). *Body ownership* refers to the perceptual status of the observer's own body, which makes bodily sensations seem unique to oneself. A positron emission tomography (PET) imaging study has indicated that this was related to activation of right posterior insula and the right frontal operculum (329). Individual differences in the report of body ownership have been associated with the cortical thickness in the somatosensory regions, the temporo-parietal junction, the intraparietal areas, and the occipitotemporal cortex (330). These studies cumulatively suggest the possibility of decreased connection between sensory body representation and brain areas involved in self-identification as the potential neural correlate of the gender-related body dysphoria experienced by many trans people.

Advances in neuroimaging techniques have enabled the possibility of quantifying and studying in a great level of detail neurobiological characteristics relating to brain structure, function, connectivity, metabolite and neurotransmitter concentrations, and nerve fiber tract integrity and activity-dependent changes in blood flow (332), all which have been explored in relation to sex and gender expression. Several studies reviewing investigations on the topic (333–338) refer to structural, functional, and metabolic brain features “exhibiting signs of masculinization or feminization”, as the result of analyzing results from research comparing transgender to cisgender groups of individuals. However, the very basis of assuming that male and female brains are categorically different is hugely controversial (339).

The existent literature reports sex differences in brain anatomy on a global scale, regarding absolute volumes (340–342). Indeed, a consistent observation in both pediatric (343, 344) and adult (341, 345–348) *in vivo* magnetic resonance imaging (MRI)-based morphometric analyses is an approximate 9%–12% greater brain size in males compared to females. Higher percentages of white matter in males in comparison to females (341, 349) are also frequently documented, as well as larger ratios of grey matter to white matter in females, even after correcting for the effect of total intracranial volume (345, 350). Studies also report sex differences in the relative sizes and shapes of regional brain structures, with the direction of the sex effect varying between regions, including the Broca's region (340), corpus callosum (351), amygdala, insula, and hippocampus (342, 352). This notion has been, however, challenged by meta-analyses that conclude the opposite (353, 354) after accounting for differences in head size. Research efforts by Joel et al. (339) into sex differences in the human brain have resurrected the idea of the “human brain mosaic”, proposed by McCarthy and Arnold four years earlier (355). Their findings, following analysis of MRI data of 1,400 individuals from four different datasets, revealed substantial overlap in the distribution of anatomical traits between men and women in all brain regions and connections examined. A wider study using data from 5,216 UK Biobank participants cautiously highlights limited sex differences in functional connectivity traits, while also referring to “considerable distributional overlap between sexes” in regional anatomical volumes, surface areas, and white matter microstructure (341). This reinforces the idea that human brains cannot, in fact, be distinctly categorized into two distinct classes, but rather that male and female brains are composed of “unique mosaics” of features, some of which are more common in one sex than the other and some that are common in both. It has been suggested that computational meta-analyses of MRI, personality, and behavioral studies have found that human brains do not exist in a “male” or “female” state (339, 356, 357). Yet, it is plausible that gendered constructions may alter gene expression through epigenetic means to produce, reinforce, or counteract endogenous sex differences (356) Given the plethora of conflicting and ambiguous results, the consistency and etiology of sex/gender differences in human brain structure and connectivity remain elusive (350).

### Neuroimaging representation of pain

Pain depends on the integration of many sensory signals, and is also influenced by biological, psychological, and social factors (358, 359). While molecular mechanisms of peripheral pain transduction have been well developed over the last few decades (360), the organization of central pain circuits is incomplete and far from being well understood. A review on pain modulatory mechanisms proposed a network involving predominantly medial and frontal cortical brain areas, combined with specific subcortical and brain stem nuclei to confirm a “key system” in the modulation of pain (361), with the prefrontal cortex likely intervening in the generation, maintenance, and integration of pain relief (361). It has also been proposed that the central processing of pain relates to the thalamus, despite evidence that pain is also controlled by cortical mechanisms in areas such as the cingulate gyrus and insular cortex (362).

Within the neuroimaging techniques, functional MRI (fMRI) including resting-state (rs-fMRI), and positron emission tomography (PET), have emerged as important tools that provide a window into pain mechanisms, highlighting the essential role of cortical and mesolimbic brain regions in modulating pain responses (363). Neuroimaging studies have shown altered brain structure and function in chronic pain states (364). Some researchers support the hypothesis that opioids and the orbitofrontal cortex modulate pain by hedonic experience, as the ventral striatum and dopamine mediate motivation drive by a painful stimulus (365). Others identify the posterior insular cortex as an important pain center, targeted in deep brain stimulation treatments to increase pain thresholds (366). Structural and diffusion tensor neuroimaging studies of patients with chronic musculoskeletal pain provide moderate and yet inconclusive evidence that microstructural white matter and regional grey matter volume in brain regions encompassing the cingulate cortex, insula, and superior frontal and temporal gyrus relate to pain intensity and sensitivity in these patients (367). Given the varying, yet complementary, information that can be obtained from different neuroimaging techniques, the complexities in assessing and managing pain, and the lack of sensitivity and specificity of individual pain biomarkers (neuroimaging included), a framework that combines different neuroimaging modalities with non-imaging data sources (i.e., actively and passively recorded bio- and psychometrics) seems to be the way forward for personalized pain diagnosis and management (368).

### Brain neuroimaging in relation to transgender identities

Neuroimaging studies suggest that, before GAHT, most transgender individuals exhibit brain features that more closely resemble their assigned gender at birth (335–338). However, some neuroimaging studies in transgender individuals have found that cortical thickness (335, 338) and subcortical volumes (337) may be more similar to those of their gender identity. This is also the case with white matter microstructure (336–338), especially in the inferior fronto-occipital white matter tract, connecting the parietal and frontal brain areas that mediate own-body perception (421), a finding confirmed by MRI studies (369). One recent MRI study comparing 24 cis men, 24 cis women, and 24 trans women (who had not undergone any GAHT) suggested a shift away from the brain anatomy typical of the cisgender men group towards the one typical of the cisgender women group in the transgender women (370).

Conflicting results exist in regard to volumes of subcortical regions like the nucleus accumbens and thalami in transgender populations compared to cisgender individuals, with some studies finding differences between both groups (371). A study that enrolled 66 transgender individuals (33 trans men and 33 trans women) reported larger putaminal volumes in the transgender group (especially trans women), compared to all cisgender participants (372).

It is noteworthy that hormone-dependent decline in brain volume has been reported to occur in postmenopausal females, or in females receiving long term anti-estrogens (373, 374). Conversely, post-menopausal females undergoing hormone replacement therapies display significantly larger volumes as compared to menopausal women without therapy, of several regions including superior/middle/Inferior frontal gyri, hypothalamus, inferior temporal gyrus, parahippocampal gyrus, hippocampus, cerebellar cortex, postcentral gyrus, precuneus, angular gyrus, supplementary motor area, superior occipital gyrus, and precentral gyrus (374).

Recent neuroimaging studies have shown functional, metabolic, and structural differences in transgender populations precisely in cortical areas that modulate pain and in sensori-motor regions (Supplementary Table S2), some of which are particularly relevant after hormonal treatment. Another study found an increase in resting-state functional connectivity between the left thalamus and the left sensori-motor cortex/putamen following GAHT in individuals that had a gender-affirming surgery, with an especially higher activation in a cluster within the subcallosal cortex (375). These results suggest that the hormonal therapy applied after gender affirming surgery may modulate functional connectivity in regions engaged in emotional and sensori-motor processes, which are part of the pain modulatory network. This suggestion is consistent with the research in preclinical models as discussed further below.

### Limitations in the analysis of the neuroimaging literature

It is difficult to establish causality between brain structural, metabolic, and functional gender differences and psychological stress factors. In other words, it is not known whether brain appearance and function are determined by psychological stress factors due to gender differences, or vice versa. Evidence from neuroimaging studies suggests that mental processes (e.g., thoughts, feelings, beliefs, volition) significantly influence various levels of brain functioning (e.g., molecular, cellular, neural circuit) and brain plasticity (376). Specifically, there is evidence that sustained stress-related factors play a role in brain structure and function (377), and that gonadal hormones influence immunity and inflammation, both of which affect the brain, directly and indirectly (378–380). A systematic review of neuroimaging studies in relation to gender (333) excludes studies on neurological diseases or aspects related with neurological outcomes, while other studies on these populations limit their analyses to sex differences assuming gender binarism and congruence between the sex assigned at birth and gender identity (381–383). It is also worth noting the complexity in the analysis of factors influencing the representation of sexes in clinical trials, even in diseases that are not supposed to be sex-related (381), and the possibility of bias in the literature given the way results are reported. Also, few original studies are truly exploratory in the sense that they explore the whole brain, while most base their analyses on hypotheses that involve only a surprisingly few brain regions. This may contribute to unconscious selection bias in any literature analysis that includes them.

### Future directions

Varying sample sizes, recruitment of convenience samples, duplicate reporting of findings in more than one publication, alternative protocols, and processing in neuroimaging are among the various potential factors responsible for inconsistencies in existing literature surrounding sex and gender-related differences in regional brain volumes (384). Considering that the surfeit of studies examining differences across brain substructures are typically underpowered and inconsistent (385), it is imperative that larger sample sizes are used when testing hypotheses regarding putative sex/gender differences in the human brain (341). Given the differential effect that age has on human brains, large-scale follow-up studies are crucial to delineate how sex/gender differences develop and change throughout the lifespan. In doing so, greater strides can be made in elucidating the neuroanatomic substrates and sex/gender-related differences in human behavior (349). Given individual differences in expectancies and conditioning effects of brain networks that modulate pain experiences, and the known effect of the psychosocial context that accompanies any therapy, further research is needed to estimate and document the degree of expectancy of nocebo responses in relation to gender identity and social factors (359, 376, 386). Neuromodulation, a technique used in functional neurosurgery to restore the brain functions by modulating the neuroanatomical and neurochemical circuits that regulate them, albeit compensating functional disorders (e.g., pain), could also have an effect in neuronal circuits underlying gender expression. Therefore, the impact of this technique on identifying neural circuitry that covaries with gender expression would also benefit from further investigation. Longitudinal neuroimaging studies on gender expression are currently limited to short-term investigations of the effects of gender affirming procedures (387–390) and cover a geographically limited sample. Long-term neuroimaging studies on gender expression across the lifespan, specifically in relation to pain, are needed.

## Can variables reflecting the role of sex be modeled in rodents?

In this final section, we raise the issue of future directions in preclinical research relevant to the understanding of pain in the context of biological variables underlying the development of sex and gender. Here, we note that much of our predictive understanding of the role of neural substrates in defining human pain biology has arisen from preclinical studies. However, the preclinical model does not presume to mirror the complexity of the biological and psychological aspects of sex and gender in humans.

As reviewed in this manuscript, there is evidence for the influence of hormones (391, 392), sex chromosome complement (393), and early life experience (394–397) in the pain phenotype expressed in human variables that are evident and controllable in the preclinical models (Supplementary Figure S1). Given the limitations posed by the study of experimental variables in humans vs. animals, we cannot discern how the impact of these variables on the human experience is impacted by each individual's sex characteristics or gender expectations. Even when sex chromosome complement and hormonal profile are discordant, such as in complete androgen insensitivity syndrome in which XY genotype is accompanied by external female phenotype (398), we do not know how much gendered expectations (e.g., induced by being raised as a girl) impact brain organization and connectivity as they are established during development. Modeling aspects of sex differences in laboratory animals such as rats and mice may with caution enable us to disentangle the impact of not only hormones and sex chromosome complement (which can be distinguished in rodents much more readily than in humans), but also to begin to understand how environmental variables and gendered expectations influence biology and behavior, including the pain phenotype and the regulation of its expression. We have empirical evidence regarding the role of hormones, chromosomes, and experience (e.g., maternal care) in rodents, but this has not been achievable in humans, leaving essential questions unanswered.

### Sexual differentiation of the rodent and human brain

The classic view of brain sexual differentiation begins with the onset of androgen production by the fetal testis during the 2nd trimester in humans and the last quarter of pregnancy in rats and mice (399). In rodent models, decades of evidence confirm the masculinizing impact of perinatal androgen exposure on the size of entire brain regions, nuclei and subnuclei, as well as the strength of projections, patterns of synaptic connectivity, and neurochemical identity of select neural populations (400, 401). These differences are established during a critical period that ends prenatally in humans and in the first few days of life in rodents. The critical period is not the same as the sensitive period, which is defined as the developmental window during which the brains of genetic females remain responsive to exogenous hormones (402). This sensitive period for hormonal modulation is believed to end *in utero* in humans but in rodents ends by the first week of life (403). By the time of birth in humans and the first week of life in rodents, hormonally induced, enduring sex differences in the brain, are firmly established, even though the brain is still highly immature, having decades left to refine in humans and almost a month in rodents. The transition from puberty to reproductive maturity is increasingly appreciated as a distinct critical/sensitive period (404, 405), but the period prior to that—toddler-to-childhood in humans and post-weaning in rodents—has been largely ignored in regard to biological origins of sex differences. In humans this period is characterized by exposure to strongly gendered expectations. It provides a speculative hypothesis, that such regional changes during this time may provide an opportunity to address the role of evolving gendered expectations in driving biological parameters such as circuit refinement, synaptic strength, and connectivity. It is clear that even in this brief overview, brain and behavioral development viz sexuality reflect multiple variable that are difficult to deconvolute (399, 406).

### Efforts to parse the relative influence of hormones, sex chromosome complement, and sex role expectations

In rodents, by comparing genetically modified mice to XY males with testes and XX females with ovaries, gonadal hormone effects can now be distinguished from sex chromosome effects. This “4 core genotype” model has revealed specific sex differences that are entirely determined by hormones, others that are entirely determined by sex chromosomes, and others that appear to be a blend of both. For example, sex differences in heat pain thresholds in neonatal mice were found to depend on sex chromosome complement and be independent of gonadal status, whereas sensitivity to mu opioid agonists depended on the presence of testes (245). The 4 core genotype model could be used much more widely to untangle sex chromosome complement vs. hormonal influences on chronic pain states, as well as on analgesic sensitivity. Further research into the influence of sex chromosomes and hormones on pain and analgesia could help us to develop insights into transgender individuals who elect to transition hormonally and/or surgically at various points in development, vs. those who do not.

### Impact of environmental context

Environmental context reflects conditions such as an enriched or deprived stimulus, milieu, and social and physical components of the environment in which the organism functions, but which induce stress to the degree that the environment challenges the repertoire of the organism's adaptive response. Aside from the acute effect of such environmental cues, the rearing environment has been noted to have prominent and enduring effects upon brain function and behavioral responsivity and engagement in humans and animals. In rodents, early environmental and psychosocial stressors can robustly influence cortical morphology and microglia resulting in adaptive responses at the level of synaptic, circuit, and neuroimmune signaling (407, 408).

An interesting example of preclinical research that might arise in this area relates to the question of being reared with gender expectations. Preclinically, the question would be phrased as assessment of the impact of sex role expectations on brain function. Although rodents develop to reproductive maturity much more rapidly than humans, they progress through comparable stages with corresponding epochs of brain development (Supplementary Figure S2). Human social systems and gendered expectations are extremely complex and clearly dependent on cultural differences. However, in preclinical models, the strongest gendered expectations are most readily modeled in animals and focus on nurturing. Male and female rats as young as 24 days old will both retrieve and nest pups (409). Juveniles sensitize to pups faster than adult virgin females, but adult males rarely do so, instead shunning pups or attempting to kill them (410, 411). Moreover, maternal behavior can be readily induced in adult virgin females by mimicking the hormonal milieu of pregnancy (412), but the same treatment in adult males is without effect. Thus, divergence from a common nurturing response in juvenile males and females to an opposite response by adults provides an opportunity to model how a sex role alters brain and behavior. This could be achieved by rewarding juvenile animals while they engage in nurturing and determining how that impacts adult brain and behavior. Thus, will a naturally occurring nurturing response in prepubertal males and females that diverges when they are adults shift when a sex role is manipulated? If so, such a manipulation could be utilized to begin to understand how gendered expectations early in life (in addition to sex chromosome complement and gonadal hormones) can influence brain and behavior and its impact upon pain processing using a rodent model.

The influence of development environment on brain connectivity and function extends beyond issues of sex and gender. In humans, early life stressors can markedly influence psychosocial reactivity, pain sensitivity and pain experience later in life (394–397). Higher socioeconomic status shows covariance of efficient cortical networks in adulthood with effects varying with early age of exposure (413). While these early experiences can have a learning component, these developmental experiences can translate into lifelong changes in cerebral function and behavior though the actions of circulating hormones. This leads to, for example, microglial activation and changes in synaptic pruning, an altering connectivity (408), or epigenetic activation and silencing of DNA. Such changes lead to long term changes affecting how our genes are read and transcribed, producing enduring effects of early experience and the environment in humans on brain function and on psychosocial function and pain behaviors much later in life (414–416).

### Future directions

It should be stressed that the preclinical model does not presume to mirror the overall complexity of the biological and psychological aspects of sex and gender in humans. However, these models allow one to systematically assess issues relevant to system function. In the context of the present review, these issues include the impact of hormone therapy on neural functioning or the response of the system to surgical interventions and to environmental stressors. The use of genetically and chromosomally modified animals in conjunction with studies in humans may provide exciting insights which can illuminate the contributions of biology, sex, gender, and the environment in which the organism is born, nurtured, and matured assuming ethical standards in these studies can be met. As noted above, behavioral investigations of the environmental context in which an animal is raised would have particular significance in studies examining brain function and biology, particularly as with the role of epigenetics in developmental expression of sex related behavioral phenotypes. Continued research into models such as the gonadectomized animal and assessing depression-like symptoms displaying a sex-difference in relationship to maternal care when young.

## NIH focus on transgender health

The National Institutes of Health makes it abundantly clear in its 2021–2025 Strategic Plan that it supports and promotes research that considers “the effects of sex and gender in study design, data collection and analysis, and dissemination of findings,” which “will help to inform the development of prevention strategies and interventions for everyone.” It goes on to elaborate that “Underserved groups—including Black, Latin origin, and Indigenous and Native American persons, Asian Americans and Pacific Islanders, and other persons of color; members of religious minorities; lesbian, gay, bisexual, transgender, and queer (LGBTQ+) persons; persons with disabilities; persons who live in rural areas; or persons otherwise adversely affected by persistent poverty or inequality—have distinct health needs and often experience disparities in health outcomes. NIH maintains that racial and ethnic minorities, rural residents, people with low incomes, SGM, and other populations experiencing health disparities should be included in all relevant research, such that there is sufficient representation of each population to conduct relevant analyses. Inclusivity in research generates more broadly applicable information and improves scientific understanding of the health and well-being of specific population groups,” (417). Despite these efforts, the enrollment reports for NIH-funded studies currently only allow reporting of 3 categories: male, female, and unknown/not reported.

As there is often no category for transgender identity in NIH enrollment reports, data on this population are often omitted or ‘hidden’ under the categories *male, female* and *unknown/not reported*. It will be critical to uniformly add categories for transgender identities (and other underserved groups such as sexual minorities) into NIH enrollment tables and to specify if “male” and “female” are meant to refer to cisgender men and women, respectively, or if “female” includes cisgender and transgender women and “male” includes cisgender and transgender men. This is essential so that the health and the health needs of this population can be studied and compared effectively throughout all NIH-funded studies.

The NIH, with the Office of Research on Women's Health (ORWH), developed the policy notice on Sex as a Biological Variable (SABV) which states that the “NIH expects that sex as a biological variable will be factored into research designs, analyses, and reporting in vertebrate animal and human studies. Strong justification from the scientific literature, preliminary data, or other relevant considerations must be provided for applications proposing to study only one sex…” (418). While the SABV policy does not pertain to gender at this point, including gender analysis in NIH research is being encouraged on many fronts. The ORWH considers gender a critical variable in its strategic plan which states: “…research focused on understanding scientifically important sex and gender differences as well as investigations of the many factors underlying the health of women are integral to NIH's mission to seek fundamental knowledge about the nature and behavior of living systems and the application of that knowledge to enhance health, lengthen life, and reduce illness and disability” (419).

In terms of pain research, the NIH Pain Consortium was established in 1996. It informed the pain research field via its flagship program announcement (PA), titled “Mechanisms, Models, Measurement, & Management in Pain Research” (420). This program announcement was first developed in 2006 and has historically expressed and currently expresses the NIH interest in pain, which considers sex and gender. For example, this PA asks for “Mechanisms that underlie gender and cultural differences.” This clearly provides a funding platform to address many of the key variables that this review has sought to illuminate in understanding pain and it mechanims and management in the transgender individual

## Summary commentary

We have sought to review the diverse literature on the intersection between sex and pain with a focus on transgender individuals. This literature has broadly focused on the diversity of the transgender population, societal factors that impact pain in trans men and women, medical interventions as with the use of gender affirmation hormone therapies, pain phenotypes in patients undergoing gender affirmation surgeries, outcome from neuroimaging assessments, and the contributions of preclinical models. Many transgender individuals seek medical interventions to change sexual features of their bodies, to better align with their internal sense of self; the interaction of these procedures with pain is clearly under studied. The emotional consequences of gender dysphoria and societal stigma present a very heavy emotional burden that has been associated with high rates of depression in trans individuals, with significant implications for pain. Our review highlights the need to further explore the disproportionate effect of pro-inflammatory social stressors together with the impact that physiological and epigenetic factors may have (if any) to adequately address their impact on mental health and influence on pain responses in transgender patients.

Differences in gender identity, surgical interventions, exogenous hormone administration, and minority stress may result in significant differences in pain risk and pain experience in transgender patients. There is limited but increasing evidence that trans individuals may acquire pain phenotypes most similar to cisgender individuals of the same gender. Accordingly, treating and managing pain in transgender patients as in cisgender patients requires appreciation of the roles played by all facets of sex, gender, medical interventions, clinical conditions, and social factors. It should be noted that we still lack data about pain phenotypes in nonbinary people and how they may perceive pain.

While current clinical reports have largely revealed favorable outcomes in patient quality of life, this review has also emphasized the need for further long-term, large-scale, research in pain mechanisms and clinical interventions in transgender individuals, and, with it, the need for a concerted effort to advance our understanding of the influence of the long term outcomes of such interventions in the physiological and emotional aspects underpinning pain responses. Similarly, more research is required to better address pain phenotypes in transgender patients undergoing hormonal and surgical interventions. Advances in epigenetics will provide exciting insights into the impact of life and environmental influences (stressors) and their interaction with sex assigned at birth and gender as it addresses the mechanism of pain, analgesia, and other psychological parameters.

Due to the complexity of the factors that may affect pain perception and considering the limitations of current research data, every individual presenting with pain, acute or chronic, requires a personalized approach to pain management that takes into account the factors considered in this review. Future research targeted at these issues is of paramount importance to human welfare in general and to trans individuals in particular.

## Supplementary material

Supplementary Figure S1

Relative contributions of variables to sex differences in brain and behavior. We have empirical evidence regarding the role of hormones, chromosomes, and experience (e.g., maternal care) in rodents, but this has not been achievable in humans, leaving an essential question unanswered.

Supplementary Figure S2

Epochs of development in humans and rodents. Hormonal effects on the developing brain are largely complete prior to birth and re-emerge at puberty, but the intervening years are characterized by dynamic brain development sensitive to modulation by environment and experience.

Supplementary Table S1

Concepts and terminology. It is appropriate that we here consider an annotated nomenclature and associated concepts relevant to commentary related to gender and sex characteristics.

Supplementary Table S2

Summary of the relevant findings of papers showing whether or not and in which way the specific brain cortical areas that intervene in pain modulation differ in relation to transgender identity. The papers showing primary analyses were identified by searching Web of Science with the terms “neuroimaging”, “transgender, *” and “cort*” on 26.01.2022 (i.e., reviews and opinion papers were excluded). MRI, magnetic resonance imaging; fMRI, functional magnetic resonance imaging; rs-fMRI, resting state fMRI; PET, positron emission tomography.
