# Supplementary material for: Pain mechanisms in the transgender individual: a review
Source: Front Pain Res (Lausanne). 2024 Mar 27;5:1241015. doi: 10.3389/fpain.2024.1241015 (PMC11004280; doi:10.3389/fpain.2024.1241015)
Supplement: Supplementary file 1 [file Table1.docx]

**Supplemental Table 1. Concepts and Terminology**

It is appropriate that we here consider an annotated nomenclature and associated concepts relevant to commentary related to gender and sex characteristics.

| *Assigned gender at birth (AGAB) or Assigned sex at birth (ASAB)* | A designation of a person’s gender/biological sex by a medical provider at birth based on visually inspecting the infant’s genitalia, which is recorded in the individual’s birth certificate or reported to a birth registry or other organization. The first country to mandate birth data collection was the United Kingdom in 1853, and the United States implemented such collection in 1902 [1]. |
| --- | --- |
| *Cisgender* | A gender modality in which one’s gender identity and assigned gender at birth (AGAB) are aligned [2, 3]. |
| *Gender* | A complex umbrella term for characteristics that relate to sociocultural designations considered to be associated with sex. Instances of gender include gender identity, gender modality, gender expression, gender roles, and gender markers, among many other representations [4]. |
| *Gender affirmation* | Any process by which gender dysphoria is decreased and/or gender euphoria is increased. Gender affirmation can be medical, social, legal, or take various other forms. Cisgender and transgender people undergo different processes of gender affirmation [5]. |
| *Gender binary, Gender binarism, or Binary gender system* | Classification of gender into two distinct groups, usually thought of as masculine and feminine. However, many other gender systems exist throughout a number of world cultures, and many binary systems do not strictly align with observed sexual dimorphism [6]. |
| *Gender dysphoria* | Psychological distress or discomfort that results from a disconnect between one’s sense of gender identity and their physical, hormonal, mental, emotional, presentational and/or social attributes and the gender assigned at birth [7, 8]. While individuals who identify as transgender and ultergender (intersex people who identify as a gender other than their assigned gender at birth, but do not feel the term “transgender” is descriptive because of being intersex) come to mind when discussing gender dysphoria, not all transgender and ultergender individuals experience gender dysphoria, and cisgender individuals can also present with it. For instance, cisgender women with polycystic ovarian syndrome may experience hirsutism and grow more noticeable facial hair [9]. In many cultures, cisgender women having more noticeable facial hair is seen as a negative trait, which can result in duress in relationship with one’s gender identity [10, 11]. |
| *Gender euphoria* | A state of intense excitement and happiness which results from one’s sense of gender identity aligning with an aspect of their physical, hormonal, mental, emotional, presentational and/or social attributes [12, 8]. For decades, gender euphoria in transgender people, especially transgender women, has been inextricably connected to ideas of “fetishism” [13], despite the fact that cisgender people, when expressing the same kind of euphoria, would also be labeled as “fetishistic” by the same mechanisms [14]. |
| *Gender identity* | A person’s self-concept of the gender category to which they belong. A person may identify with one gender identity label, multiple gender identity labels, or no gender identity labels [3]. Additionally, an individual may change gender identity labels over the life course [3]. Non-human animals are not considered to have a gender identity. |
| *Gender incongruence* | A long-term pattern of gender dysphoria, which is typically unique to transgender and ultergender persons (see below for definition of ultragender), in which one’s assigned gender at birth (or gender of rearing, the gender they were raised as, etc.) differs from their gender identity [15, 16]. |
| *Gender marker* | A marker which indicates gender on a given document (such as a driver’s license, birth certificate, etc.) or in a given database (such as in birth registry, an electronic health record, etc.). [3]. |
| *Gender marker on health insurance* | The gender marker which appears on one’s health insurance card, is registered with their health insurance company, or otherwise appears in a form or document associated with health insurance [3]. |
| *Gender modal*ity | The relationship between one’s gender identity and assigned gender at birth (AGAB), or more broadly, the gender that one was raised or reared as [3, 2]. |
| *Gender transition* | Any of various social, legal and/or medical processes by which an individual moves from one form of gender expression or gendered physicality to another. Often shortened to *transition* [17]. |
| *Gender-affirming hormone therapy* (GAHT) | Therapeutic use of exogenous hormones for the primary purpose of gender affirmation. Typically, GAHT involves some use of anti-androgens, estradiol, progesterone, and/or testosterone by one or multiple routes. Cisgender people and transgender people access GAHT in some way, with menopausal cisgender women and cisgender men with sexual dysfunction being two of the groups that utilize GAHT [18]. |
| *Gender-affirming medical procedure* | Any medical procedures performed for the primary purpose of gender affirmation. It is/can be applied irrespectively to cisgender and transgender individuals. |
| *Gender-affirming surgery (GAS)* | Surgical procedures, briefly reviewed in this scoping review used for the primary purpose of gender affirmation. |
| *Hermaphrodite* | An intersex phobic slur to refer to individuals with ambiguous external genitalia, a perceived blurring between the socially (mis)understood to be “masculine” and “feminine” features, and the presence of either testes or ovaries. In reproductive biology, a hermaphrodite is an organism that has both kinds of reproductive organs and can produce both gametes associated with male and female sexes [19]. Of note, the term hermaphrodite, and its derivatives (such as hermaphroditic) are appropriately applied to some non-mammal biological species (e.g., flowering plants, some insects, and gastropods among others). |
| *Intersex* | An umbrella term referring to variations in sex characteristics which, while present congenitally, may become apparent at any period of one’s life course. Intersex variations are usually anatomical, genetic, or hormonal in nature. Further, just because a particular variation may be considered intersex in some cases, does not mean it is intersex in all cases. Intersex people are usually coercively assigned (oftentimes via intersex genital mutilation) as “male” or “female”, meaning that intersex is usually not an assigned gender at birth (AGAB). Intersex as a term should only be used when referring to humans [20, 21]. |
| *Ipsogender* | A label describing when an intersex person’s gender modality is cisgender [3]. |
| *Legal gender* | A social and legal construction often used to refer to gender markers (erroneously labeled as ‘sex’) on identity documents. e.g., birth certificates, driver’s licenses, and passports. Because of the difficulties involved in changing one’s gender marker on any of these documents, it is possible that an individual has two or three different “legal” genders [22]. |
| *Legal name* | A legal construction which refers to the name or names appearing on identity documents. Because of difficulties involved in changing one’s name on these documents, it is possible for an individual to have multiple “legal” names. The fact that one’s name on identity documents does not match their actual name, can be equally seen for cisgender and transgender people. (37, 405) |
| *Name* | Occasionally also referred to as a ‘name to use’ or a ‘name used’; one’s name is the primary identifier that an individual uses and which is used to refer to that individual. Importantly, an individual’s name is just their name, it is not ‘chosen’, ‘preferred’, ‘affirmed’, etc. [3, 23]. |
| *Neurosexism or Neurological sexism* | The term reflects the assumption that gender differences perceived in character and behavior are caused by observed differences in brain structure or activity [24]. It has been suggested that the “brain sex” model is a self-fulfilling prophecy model [25, 26]; likewise, computational meta-analyses of MRI, personality, and behavioral studies have found that human brains do not exist in a “male” or “female” state [27-29]. Yet, it is plausible that gendered constructions may alter gene expression through epigenetic means to produce, reinforce, or counteract endogenous sex differences [28]. Observed structural differences have been used to justify sexism [26], homophobia [30], and transphobia [30-32], which perpetuates so-called character and behavior differences. |
| *Nonbinary* | An umbrella term referring to any of various gender identities outside of the binary of male and female; (2) A specific gender identity term which is not strictly male or female and therefore does not fit within the gender binary. Nonbinary people may describe their identities in a number of ways, including, but not limited to (a) being exclusive or specific to one’s culture, intersex status, or neurotype, (b) being fluid or fluctuating, (c) being multiple or multiplicative, (d) being an absence of gender or a state of non-genderedness, (e) overlapping, ambiguous, or between genders, (f) partially a gender or genders, and (g) indescribable or unnamable. Note that “nonbinary” (no hyphen) is more common in the United States, while “non-binary” (hyphenated) is more common in the United Kingdom [33]. |
| *Pronouns* | The set of pronouns that an individual uses. In English, the most common pronouns used by individuals are she/her, he/him, and they/them, although a number of neopronoun sets also exist, such as ze/hir and ey/em. Grammatically, a pronoun set in English includes a subject pronoun (*she/he/they*), object pronoun (*her/his/them*), a possessive determiner (*her/his/their*), possessive pronoun (*hers/his/theirs*), and a reflexive pronoun (*herself/himself/themself*). Many non-English languages also have gendered pronouns (and gender-neutral neo-pronouns), while 57% of world languages do not have gendered pronouns [34]. However, many of those languages have other forms of gender signification, such as titles, prefixes, and suffixes. Importantly, an individual’s pronouns are just their pronouns, they are not ‘chosen,’ ‘preferred,’ etc. Not using an individual’s correct pronouns is typically considered to be a form of misgendering and has been tied to higher levels of anxiety and depression. |
| *Pubertal inhibition therapy* | Any of various therapies intended to delay pubertal change(s). Cisgender and transgender youth both utilize pubertal inhibition therapy, which has been utilized since the 1950s, initially for use in precocious puberty [35]. Because Gender-affirming hormone therapy (GAHT) is often restricted to those above age 16 (occasionally above 14), pubertal inhibition therapy may be provided as a stop-gap measure following the onset of Tanner Stage 2. |
| *Sex* | A complex label used to describe the totality of distinguishable characteristics of an organism, including its reproductive structure, functions, phenotype, and genotype [3, 36]. In practice, the usefulness of sex as a metric depends heavily on the number of sexes observed in a given species, the degree of sexual differentiation within that species, and the probability of encountering one or more characteristics which do not fit the multimodal distribution(s) presented. In non-human organisms, it is recommended that one avoid using ‘sex’ unless a specific process of how sexing was performed is outlined by the researcher (such as *sexing by gonad presence*, *sexing by karyotype*, *sexing by dry weight*, etc.). In humans, it is recommended to use *assigned gender at birth* when referring to a (shortly after birth) assignment of a gender marker on a birth certificate or other birth record. |
| *Sex characteristics* | Characteristics considered to be associated with sex in some form or manner. Sex characteristics are often *sexual* or *reproductive* in nature, having direct relationships to processes of sexual intercourse and various reproductive processes, or nonsexual and nonreproductive, such as differing coloration (such as in peacocks/peahens) or different average size (such as in anole lizards). Sex characteristics in humans are often *gendered*, having gendered expectations related to them. (37, 416) |
| *Sex dyadicity* | The state of being intersex or non-intersex (also called *perisex* or *endosex*). |
| *Sex for clinical use (SFCU)* | A sex designation tied to a specific clinical situation, based on how electronic health records currently function; for instance, a clinical test may have “male” or “female” reference ranges, and choice of one represents a sex for clinical use datum [23]. |
| *Sexual dimorphism* | A state in which a species’ primary and secondary sex characteristics are highly associated in two primary forms. Note that *dimorphism* does not indicate a binary, as a binary means that only two combinations are possible (such as a ‘0’ or ‘1’ in binary computer code), while dimorphic means that two combinations are more common, but that other combinations are possible (such as in a bimodal distribution) [37]. |
| *Transgender* | (1) A gender modality in which one’s gender identity and assigned gender at birth (AGAB) are not aligned [3, 2]; (2) An umbrella term referring to gender identity, gender expression, etc. which is considered to cross sociocultural boundaries of what a particular gender can be. *Transgender* may be shortened as “trans”. |
| *Ultergender* | A putative label describing when an intersex person’s gender modality is transgender [3]. |

**REFERENCES**

1. Brumberg HL, Dozor D, Golombek SG. History of the birth certificate: from inception to the future of electronic data. J Perinatol. 2012;32(6):407-11. doi:10.1038/jp.2012.3.

2. Ashley F. “Trans” Is My Gender Modality: A Modest Terminological Proposal. <https://www.florenceashley.com/uploads/1/2/4/4/124439164/florence_ashley_trans_is_my_gender_modality.pdf>. 2021. Accessed 21 April 2021.

3. Kronk CA, Everhart AR, Ashley F, Thompson HM, Schall TE, Goetz TG et al. Transgender data collection in the electronic health record: Current concepts and issues. J Am Med Inform Assoc. 2022;29(2):271-84. doi:10.1093/jamia/ocab136.

4. Council of Europe. Sex and gender. 2023. https://www.coe.int/en/web/gender-matters/sex-and-gender. Accessed April 3, 2023.

5. Association of American Medical Colleges. What is gender-affirming care? Your questions answered. https://www.aamc.org/news/what-gender-affirming-care-your-questions-answered. Accessed April 12, 2022.

6. Wikipedia contributors. Gender binary. *Wikipedia, The Free Encyclopedia*. <https://en.wikipedia.org/w/index.php?title=Gender_binary&oldid=1181170738>. Accessed  November 2023 18:15 UTC.

7. Ashley F. The Misuse of Gender Dysphoria: Toward Greater Conceptual Clarity in Transgender Health. Perspect Psychol Sci. 2021;16(6):1159-64. doi:10.1177/1745691619872987.

8. Badgley J. The Gender Dysphoria Bible. 2021. <https://genderdysphoria.fyi/gdb.pdf>. Accessed 12 March 2021.

9. Spritzer PM, Barone CR, Oliveira FB. Hirsutism in Polycystic Ovary Syndrome: Pathophysiology and Management. Curr Pharm Des. 2016;22(36):5603-13. doi:10.2174/1381612822666160720151243.

10. Chalabi M. Female facial hair: if so many women have it, why are we so deeply ashamed? The Guardian News Website. 2017. <https://www.theguardian.com/fashion/2017/nov/30/female-facial-hair-if-so-many-women-have-it-why-are-we-so-deeply-ashamed>. Accessed 21 March 2021.

11. Liu M, Murthi S, Poretsky L. Polycystic Ovary Syndrome and Gender Identity. Yale J Biol Med. 2020;93(4):529-37. PMID: 33005117; PMCID: PMC7513432.

12. Bradford NJ, Rider GN, Spencer KG. Hair removal and psychological well-being in transfeminine adults: associations with gender dysphoria and gender euphoria. J Dermatolog Treat. 2021;32(6):635-42. doi:10.1080/09546634.2019.1687823.

13. Serano J. Autogynephilia: A scientific review, feminist analysis, and alternative ‘embodiment fantasies’ model. *Sociol Re*. 2020;68:763-78. doi:10.1177/0038026120934690.

14. Moser C. Autogynephilia in women. J Homosex. 2009;56(5):539-47. doi:10.1080/00918360903005212.

15. Claahsen-van der Grinten H, Verhaak C, Steensma T, Middelberg T, Roeffen J, Klink D. Gender incongruence and gender dysphoria in childhood and adolescence-current insights in diagnostics, management, and follow-up. Eur J Pediatr. 2021;180(5):1349-57. doi:10.1007/s00431-020-03906-y.

16. Fernández Rodríguez M. Gender Incongruence is No Longer a Mental Disorder. J Ment Health Clin Psychol. 2018;2:6-8. doi:10.29245/2578-2959/2018/5.1157.

17. Wikipedia contributors. Gender transition. *Wikipedia, The Free Encyclopedia*. <https://en.wikipedia.org/w/index.php?title=Gender_transition&oldid=1158989337>. Accessed 8 November 2023.

18. Irving A, Lehault WB. Clinical pearls of gender-affirming hormone therapy in transgender patients. Ment Health Clin. 2017;7(4):164-7. doi:10.9740/mhc.2017.07.164.

19. Minelli A, Fusco G. The biology of reproduction. Cambridge, United Kingdom ; N.Y., NY: Cambridge University Press; 2019.

20. Rosenwohl-Mack A, Tamar-Mattis S, Baratz AB, Dalke KB, Ittelson A, Zieselman K et al. A national study on the physical and mental health of intersex adults in the U.S. PLoS One. 2020;15(10):e0240088. doi:10.1371/journal.pone.0240088.

21. Carpenter M. Intersex Variations, Human Rights, and the International Classification of Diseases. Health Hum Rights. 2018;20(2):205-14. PMID: 30568414; PMCID: PMC6293350.

22. Wikipedia contributors. Legal gender. *Wikipedia, The Free Encyclopedia*. <https://en.wikipedia.org/w/index.php?title=Legal_gender&oldid=1166320276>. Accessed 8 November 2023.

23. McClure RC, Macumber CL, Kronk C, Grasso C, Horn RJ, Queen R et al. Gender harmony: improved standards to support affirmative care of gender-marginalized people through inclusive gender and sex representation. J Am Med Inform Assoc. 2022;29(2):354-63. doi:10.1093/jamia/ocab196.

24. Eliot L. Neurosexism: the myth that men and women have different brains. Nature. 2019;566.453-454. doi: https://doi.org/10.1038/d41586-019-00677-x

25. Begley S. Why Parents May Cause Gender Differences in Kids. Newsweek. 2009. https://www.newsweek.com/why-parents-may-cause-gender-differences-kids-79501

26. Rivers C BR. Neurosexism: Brains, Gender and Tech. Re/code (Re/code is a CNBC partner site). 2014. <http://web.archive.org/web/20141013041748/https://www.recode.net/2014/10/09/neurosexism-brains-gender-and-tech/>.

27. Joel D, Berman Z, Tavor I, Wexler N, Gaber O, Stein Y et al. Sex beyond the genitalia: The human brain mosaic. Proc Natl Acad Sci U S A. 2015;112(50):15468-73. doi:10.1073/pnas.1509654112.

28. Massa MG, Correa SM. Sexes on the brain: Sex as multiple biological variables in the neuronal control of feeding. Biochim Biophys Acta Mol Basis Dis. 2020;1866(10):165840. doi:10.1016/j.bbadis.2020.165840.

29. Eliot L, Ahmed A, Khan H, Patel J. Dump the "dimorphism": Comprehensive synthesis of human brain studies reveals few male-female differences beyond size. Neurosci Biobehav Rev. 2021;125:667-97. doi:10.1016/j.neubiorev.2021.02.026.

30. Grijseels D. Why biological studies on queer people do more harm than good. 2020. <https://massivesci.com/articles/lgbt-science-gender-identity-tolerance-acceptance>. Accessed 21 Mar 2020.

31. Swartz A. “Are you really trans?”: The Problem with Trans Brain Science. IJFAB Blog. 2018. <http://www.ijfab.org/blog/2018/07/3694/>. Accessed 6 May 2021.

32. Cornel T. Dear Neuroscience: Stop Trying to “Fix” Diversity. The Semipermeable Membrane. 2020. https://hpsns.hypotheses.org/1050. Accessed April 3, 2023.

33. Chew D, Tollit MA, Poulakis Z, Zwickl S, Cheung AS, Pang KC. Youths with a non-binary gender identity: a review of their sociodemographic and clinical profile. Lancet Child Adolesc Health. 2020;4(4):322-30. doi:10.1016/S2352-4642(19)30403-1.

34. Wade L. Map of the Week: 57% of Languages Do Not Have Gendered Pronouns. <https://thesocietypages.org/socimages/2014/10/11/map-of-the-week-57-of-languages-do-not-have-gendered-pronouns/>. 2014. Accessed 21 March 2022.

35. Mahfouda S, Moore JK, Siafarikas A, Zepf FD, Lin A. Puberty suppression in transgender children and adolescents. Lancet Diabetes Endocrinol. 2017;5(10):816-26. doi:10.1016/S2213-8587(17)30099-2.

36. Miyagi M, Guthman EM, Sun SED. Transgender rights rely on inclusive language. Science. 2021;374(6575):1568-9. doi:10.1126/science.abn3759.

37. Northern Arizona University. Sexual dimorphism. Northern Arizona University, Flagstaff, AZ. Sexual Dimorphism (nau.edu). Accessed November 11, 2023.
