# Supplementary material for: Pain mechanisms in the transgender individual: a review
Source: Front Pain Res (Lausanne). 2024 Mar 27;5:1241015. doi: 10.3389/fpain.2024.1241015 (PMC11004280; doi:10.3389/fpain.2024.1241015)
Supplement: Supplementary file 2 [file Table2.docx]

**Supplemental Table 2. Summary of the relevant findings of papers showing whether or not and in which way the specific brain cortical areas that intervene in pain modulation differ in relation to transgender identity.** The papers showing primary analyses were identified by searching Web of Science with the terms “neuroimaging”, “transgender, *” and “cort*” on 26.01.2022 (i.e., reviews and opinion papers were excluded).

Legend: MRI: magnetic resonance imaging, fMRI: functional magnetic resonance imaging, rs-fMRI: resting state fMRI, PET: positron emission tomography

| **Reference** | **Imaging technique** | | **Sample** | **Main findings** |
| --- | --- | --- | --- | --- |
| [1] | rs-fMRI | | 22 trans men pre and post-hormonal treatment, 19 cisgender people | Increase trans men’s cortical insular cortex and fractional anisotropy after hormonal treatment |
| [2] | MEG | | 8 trans men pre hormonal treatment, 8 cisgender females | Trans men’s activation reduced in the supramarginal gyrus and secondary somatosensory cortex, and increased at the temporal pole |
| [3] | MRI | 29 trans men post-hormonal treatment, 34 cisgender females, 28 cisgender males | | Intermediate pattern in trans men’s parietal cortex |
| [4] | fMRI | 20 transgender people pre hormonal treatment, 20 cisgender people | | Trans men had higher activation than cisgender female in the bilateral precuneus, and in trans women had higher activation than in cisgender male in the precuneus, as well as in the posterior cingulate gyrus, the angular gyrus, and the lateral occipital cortices |
| [5] | fMRI | 19 trans women pre- and 23 trans women post-hormonal treatment | | amygdala-prefrontal co-activation in trans women post-treatment |
| [6] | MRI, fMRI | 18 trans men pre- and post-hormonal treatment | | Decreased gray matter volume, increased levels of testosterone in Broca’s and Wernicke’s areas; and increased functional connectivity between the two brain regions post-treatment |
| [7] | MRI | 24 trans women and 40 trans men pre- and post-hormonal treatment, 19 cisgender people | | Trans men’s mesial temporal and insular cortices increased, trans women’s widespread cortical thinning |
| [8] | PET | 7 trans women and 11 trans men pre- and post-hormonal treatment, 17 cisgender people | | Trans men’s levels of monoamine oxidase A (MAO-A) reduced in middle frontal cortex, anterior cingulate cortex, medial cingulate cortex, insula, amygdala, and hippocampus |
| [9] | PET | 33 transgender pre- and post-hormonal treatment | | Increased SERT in trans men binding in amygdala, caudate, putamen and median raphe nucleus, in trans women decreases in SERT binding in insula, anterior, and mid-cingulate cortex |
| [10] | fMRI | 30 transgender people post-hormonal treatment, 30 cisgender people | | Greater limbic involvement in transgender people |
| [11] | MRI | 28 trans men post-hormonal treatment, 34 cisgender males and 34 cisgender females | | Thicker mid-frontal, precuneal-parietal and lingual cortex in trans men, and weaker functional connections from the pregenual anterior cingulate to the insular cortex |
| [12] | MRI, rs-fMRI | 27 trans women and 40 trans men post-hormonal treatment, 70 cisgender males and 70 cisgender females | | Greater cortical thickness and weaker structural and functional connections in the anterior cingulate-precuneus and right occipito-parietal cortex in transgender people |
| [13] | rs-fMRI | 19 trans women and 19 trans men post-hormonal treatment, 21 cisgender males and 20 cisgender females | | Low frequency fluctuations and regional homogeneity differ between transgender and cisgender people in the frontal cortex, medial temporal lobe, and cerebellum |
| [14] | MRI | 19 trans women and 19 trans men post-hormonal treatment, 21 cisgender males and 20 cisgender females | | Decreased trans women’s primary sensori-motor regions and increased trans men’s parietal cortex compared to cisgender people |
| [15] | rs-fMRI | 18 trans women pre- and post-hormonal treatment | | Increase in the rs-functional circuit between the left thalamus and the left sensorimotor cortex after hormonal treatment |
| [16] | MRI | 18 trans women pre- and post-hormonal treatment | | Decrease in left superior frontal gyrus, the left middle temporal gyrus, the right precuneus, the right superior temporal gyrus, and the right pars opercularis after hormonal treatment |
| [17] | MRI | 14 trans women and 25 trans men pre- and post-hormonal treatment | | Decrease trans women’s hippocampal region and increase trans women’s ventricles post-treatment |
| [18] | fMRI | Trans women and trans men, cisgender males and cisgender females | | Trans men’s insula similar to that of cisgender groups |
| [19] | MRI | 20 trans women pre- and 20 trans women post-hormonal treatment, 20 cisgender males and 20 cisgender females | | Trans women’s insula lower than in cisgender females |
| [20] | MRI | 54 trans men pre- and 38 trans women post-hormonal treatment, 41 cisgender females and 44 cisgender males | | Occipito-parietal cortex and sensory motor cortex reduced in transgender people |

**REFERENCES**

1. Burke SM, Manzouri AH, Dhejne C, Bergström K, Arver S, Feusner JD et al. Testosterone Effects on the Brain in Transgender Men. Cereb Cortex. 2018;28(5):1582-96. doi:10.1093/cercor/bhx054.

2. Case LK, Brang D, Landazuri R, Viswanathan P, Ramachandran VS. Altered White Matter and Sensory Response to Bodily Sensation in Female-to-Male Transgender Individuals. Arch Sex Behav. 2017;46(5):1223-37. doi:10.1007/s10508-016-0850-z.

3. Collet S, Bhaduri S, Kiyar M, T'Sjoen G, Mueller S, Guillamon A. Characterization of the H-1-MRS metabolite spectra in transgender men with gender dysphoria and cisgender people

. J Clin Med. 2021;10(12). doi:10.3390/jcm10122623.

4. Fisher AD, Ristori J, Castellini G, Cocchetti C, Cassioli E, Orsolini S et al. Neural Correlates of Gender Face Perception in Transgender People. J Clin Med. 2020;9(6). doi:10.3390/jcm9061731.

5. Grannis C, Leibowitz SF, Gahn S, Nahata L, Morningstar M, Mattson WI et al. Testosterone treatment, internalizing symptoms, and body image dissatisfaction in transgender boys. Psychoneuroendocrinology. 2021;132:105358. doi:10.1016/j.psyneuen.2021.105358.

6. Hahn A, Kranz GS, Sladky R, Kaufmann U, Ganger S, Hummer A et al. Testosterone affects language areas of the adult human brain. Hum Brain Mapp. 2016;37(5):1738-48. doi:10.1002/hbm.23133.

7. Kilpatrick LA, Holmberg M, Manzouri A, Savic I. Cross sex hormone treatment is linked with a reversal of cerebral patterns associated with gender dysphoria to the baseline of cisgender controls. Eur J Neurosci. 2019;50(8):3269-81. doi:10.1111/ejn.14420.

8. Kranz GS, Spies M, Vraka C, Kaufmann U, Klebermass EM, Handschuh PA et al. High-dose testosterone treatment reduces monoamine oxidase A levels in the human brain: A preliminary report. Psychoneuroendocrinology. 2021;133:105381. doi:10.1016/j.psyneuen.2021.105381.

9. Kranz GS, Wadsak W, Kaufmann U, Savli M, Baldinger P, Gryglewski G et al. High-Dose Testosterone Treatment Increases Serotonin Transporter Binding in Transgender People. Biol Psychiatry. 2015;78(8):525-33. doi:10.1016/j.biopsych.2014.09.010.

10. Majid DSA, Burke SM, Manzouri A, Moody TD, Dhejne C, Feusner JD et al. Neural Systems for Own-body Processing Align with Gender Identity Rather Than Birth-assigned Sex. Cereb Cortex. 2020;30(5):2897-909. doi:10.1093/cercor/bhz282.

11. Manzouri A, Kosidou K, Savic I. Anatomical and Functional Findings in Female-to-Male Transsexuals: Testing a New Hypothesis. Cereb Cortex. 2017;27(2):998-1010. doi:10.1093/cercor/bhv278.

12. Manzouri A, Savic I. Possible Neurobiological Underpinnings of Homosexuality and Gender Dysphoria. Cereb Cortex. 2019;29(5):2084-101. doi:10.1093/cercor/bhy090.

13. Mueller SC, Wierckx K, Jackson K, T'Sjoen G. Circulating androgens correlate with resting-state MRI in transgender men. Psychoneuroendocrinology. 2016;73:91-8. doi:10.1016/j.psyneuen.2016.07.212.

14. Mueller SC, Wierckx K, T'Sjoen G. Neural and Hormonal Correlates of Sexual Arousal in Transgender Persons. J Sex Med. 2020;17(12):2495-507. doi:10.1016/j.jsxm.2020.08.021.

15. Schneider MA, Spritzer PM, Minuzzi L, Frey BN, Syan SK, Fighera TM et al. Effects of Estradiol Therapy on Resting-State Functional Connectivity of Transgender Women After Gender-Affirming Related Gonadectomy. Front Neurosci. 2019;13:817. doi:10.3389/fnins.2019.00817.

16. Schneider MA, Spritzer PM, Suh JS, Minuzzi L, Frey BN, Schwarz K et al. The Link between Estradiol and Neuroplasticity in Transgender Women after Gender-Affirming Surgery: A Bimodal Hypothesis. Neuroendocrinology. 2020;110(6):489-500. doi:10.1159/000502977.

17. Seiger R, Hahn A, Hummer A, Kranz GS, Ganger S, Woletz M et al. Subcortical gray matter changes in transgender subjects after long-term cross-sex hormone administration. Psychoneuroendocrinology. 2016;74:371-9. doi:10.1016/j.psyneuen.2016.09.028.

18. Smith E, Junger J, Pauly K, Kellermann T, Neulen J, Neuschaefer-Rube C et al. Gender incongruence and the brain - Behavioral and neural correlates of voice gender perception in transgender people. Horm Behav. 2018;105:11-21. doi:10.1016/j.yhbeh.2018.07.001.

19. Spizzirri G, Duran FLS, Chaim-Avancini TM, Serpa MH, Cavallet M, Pereira CMA et al. Grey and white matter volumes either in treatment-naïve or hormone-treated transgender women: a voxel-based morphometry study. Sci Rep. 2018;8(1):736. doi:10.1038/s41598-017-17563-z.

20. Wang Y, Khorashad BS, Feusner JD, Savic I. Cortical Gyrification in Transgender Individuals. Cereb Cortex. 2021;31(7):3184-93. doi:10.1093/cercor/bhaa412.
