# Supplementary material for: Pain mechanisms in the transgender individual: a review
Source: Front Pain Res (Lausanne). 2024 Mar 27;5:1241015. doi: 10.3389/fpain.2024.1241015 (PMC11004280; doi:10.3389/fpain.2024.1241015)

Supplemental Figure 1: Relative contributions of variables to sex differences in brain and behavior. We have empirical evidence regarding the role of hormones, chromosomes, and experience (e.g., maternal care) in rodents, but this has not been achievable in humans, leaving an essential question unanswered.

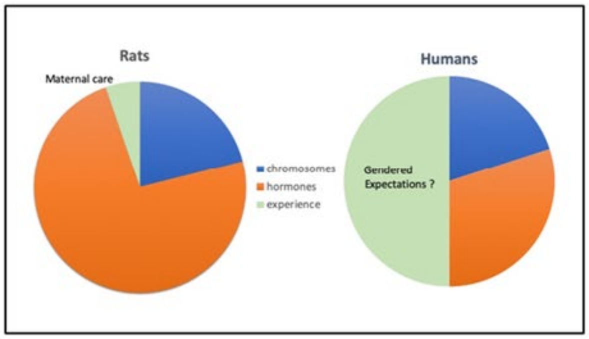

Supplement: Supplementary file 3 [file Image1.pdf]
