# Supplementary material for: Pain mechanisms in the transgender individual: a review
Source: Front Pain Res (Lausanne). 2024 Mar 27;5:1241015. doi: 10.3389/fpain.2024.1241015 (PMC11004280; doi:10.3389/fpain.2024.1241015)

Supplemental Figure 2: Epochs of development in humans and rodents. Hormonal effects on the developing brain are largely complete prior to birth and re-emerge at puberty, but the intervening years are characterized by dynamic brain development sensitive to modulation by environment and experience.

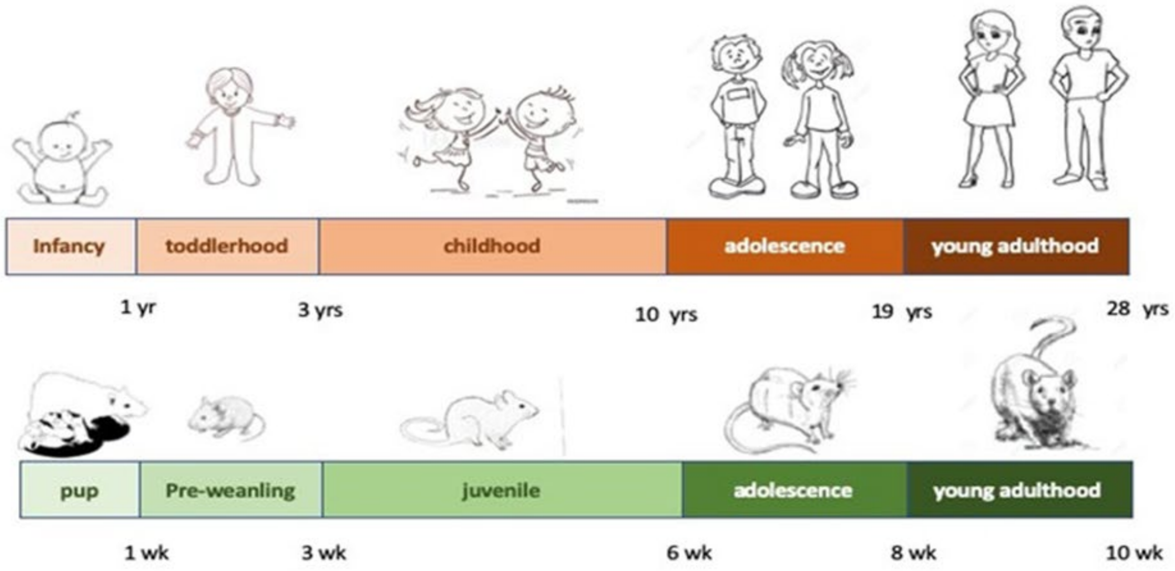

Supplement: Supplementary file 4 [file Image2.pdf]
